# Underwater sonar image detection: A combination of non-local spatial information and quantum-inspired shuffled frog leaping algorithm

**DOI:** 10.1371/journal.pone.0177666

**Published:** 2017-05-18

**Authors:** Xingmei Wang, Shu Liu, Zhipeng Liu

**Affiliations:** 1 College of Computer Science and Technology, Harbin Engineering University, Harbin, Heilongjiang Province, P.R. China; 2 Brainnetome Center, Institute of Automation, Chinese Academy of Sciences, Beijing, PR China; 3 Key Lab of Intelligent Information Processing of CAS, Institute of Computing Technology, Chinese Academy of Sciences, Beijing, PR China; Nanjing Normal University, CHINA

## Abstract

This paper proposes a combination of non-local spatial information and quantum-inspired shuffled frog leaping algorithm to detect underwater objects in sonar images. Specifically, for the first time, the problem of inappropriate filtering degree parameter which commonly occurs in non-local spatial information and seriously affects the denoising performance in sonar images, was solved with the method utilizing a novel filtering degree parameter. Then, a quantum-inspired shuffled frog leaping algorithm based on new search mechanism (QSFLA-NSM) is proposed to precisely and quickly detect sonar images. Each frog individual is directly encoded by real numbers, which can greatly simplify the evolution process of the quantum-inspired shuffled frog leaping algorithm (QSFLA). Meanwhile, a fitness function combining intra-class difference with inter-class difference is adopted to evaluate frog positions more accurately. On this basis, recurring to an analysis of the quantum-behaved particle swarm optimization (QPSO) and the shuffled frog leaping algorithm (SFLA), a new search mechanism is developed to improve the searching ability and detection accuracy. At the same time, the time complexity is further reduced. Finally, the results of comparative experiments using the original sonar images, the UCI data sets and the benchmark functions demonstrate the effectiveness and adaptability of the proposed method.

## Introduction

Currently, underwater detection technology is used extensively for seabed surveys, salvage operations, pipe-line inspections, underwater positioning, and numerous other marine applications. Sonar is one of the most important detection equipment, which can analyze the underwater objects through acoustic intensity, frequency, and phase. Underwater sonar image contains three types of regions: object-highlight, shadow and background region. The object-highlight region originates from acoustic wave reflection from an object. The shadow region stems from a lack of acoustic backscatter behind the object. The remaining information consists of the so-called “background region” [1]. Due to the complexity of the underwater environment, sand, stones and animals in sea water can cause the gray values of the background region to be close to those of object-highlight region and the shadow region. Underwater sonar image contains a large amount of ambient noise and speckle noise from the surface or sea floor [2]. Therefore, removing these noises is an indispensable step in the process of underwater sonar image processing.

The spatial information include local spatial information and non-local spatial information, which are often used to remove image noise. Local spatial information uses only the neighborhood window of every pixel. Songcan Chen et al. [3] who define the mean spatial information and median spatial information by computing the mean value and median value in the neighborhood window of every pixel. Then, they were integrated into the objective function of the fuzzy C-means (FCM) clustering algorithm to eliminate the influence of noise in the image. L. Szilágyi et al. [4] presented the idea of linearly-weighted local spatial information. For each pixel, the spatial information was obtained from that pixel and its neighboring pixels. Subsequently, nonlinearly weighted local spatial information was proposed, which was determined not only from the original image and the gray values within the neighborhood window around each pixel but also from the spatial coordinates [5]. On this basis, Stelios Krinidis et al. [6] proposed a novel local spatial information called the local spatial fuzzy factor, which incorporated both local spatial distance and local gray information in a fuzzy way to balance noise and preserve the image detail. Hui Zhang et al. [7] incorporated local spatial information into the Gaussian Mixture Model, which greatly reduced the influence of noise during the process of image segmentation. Meanwhile, Long Thanh Ngo et al. [8] introduced an approach that exploits local spatial information between a pixel and its neighbors to remove noise from satellite images. However, there are many pixels that have similar neighborhood configuration with each pixel in an image [9]. Compared with using adjacent pixels, it is more reasonable to utilize pixels with a similar neighborhood configuration to obtain spatial information for a given pixel. This type of information is referred to as non-local spatial information. In the process of computing non-local spatial information, two windows must be defined: the search window and the neighborhood window. Using the neighborhood windows of two pixels, the weighted Euclidean distance between two pixels can be computed, which can describe the similarity between those two pixels. By computing the weighted Euclidean distance between every pixel and the center pixel in the search window, the weight of every pixel in the search window can be obtained. The greater the distance, the smaller the weight of this pixel. The weighted average value of all the pixels in the search window is defined as the non-local spatial information. Jie Feng et al. [10] proposed a method to remove noise in radar images using non-local spatial information. Feng Zhao et al. [11, 12] utilized non-local spatial information to reduce the noise in medical images.

Underwater sonar images have amounts of noise and relatively weak contrast. Although the non-local spatial information can solve the noise problem effectively, the selection of the filtering degree parameter greatly affects the denoising result when computing non-local spatial information. The mean value or median value of the weighted Euclidean distance between each pixel and its center pixel is generally used as the filtering degree parameter [9]. Through an analysis of comparative experiment results, when the filtering degree parameter is selected as the mean value or the median value, there are the filtering degree parameter values over a wide range. When the filtering degree parameter is relatively large, detail information is lost in the sonar image, especially boundary information. In contrast, when the filtering degree parameter is relatively small, the noise is not removed effectively in the sonar image. To solve this problem that non-local spatial information cannot remove noise effectively in sonar image because of inappropriate filtering degree parameter, a novel filtering degree parameter is proposed in this paper. Two threshold values are set to remove relatively large and small parameters according to characteristics of sonar image in experiment results. Through two threshold values, the denosing performance of non-local spatial information is improved to some extent. The proposed filtering degree parameter is conducive to the subsequent detection of underwater objects.

The purpose of object detection is to extract the object-highlight region and shadow region from the complex background region while preserving the original edge information of sonar image as much as possible [13]. At present, research on image detection technology is very extensive, but less work has been conducted in the field of object detection from underwater sonar images [14]. In particularly, the heuristic optimization algorithms are a new important research field in sonar image detection.

In recent years, heuristic optimization algorithms have gradually attracted more and more attention both domestically and abroad. Many researches have shown that these algorithms can solve numerous complicated computing problems. Among them, the genetic algorithm (GA) [15], the particle swarm optimization (PSO) [16] and the shuffled frog leaping algorithm (SFLA) [17] are the most commonly used. In 2003, Eusuff and Lansey proposed the SFLA, which combines the advantages of the Memetic Algorithm (MA) in terms of genetic evolution and the PSO algorithm in terms of social behavior [18, 19]. SFLA has a few parameters and good searching ability. Its structure is very simple and easy to implement. Moreover, its performance is better than GA and PSO. Therefore, SFLA has promising development and application prospects. Xiaodan Zhang et al. [20] developed power control algorithm in cognitive radio system based on a modified SFLA. Fan Tanghuai et al. [21] proposed a strategy to improve the learning process of frogs and extend the learning diversity of frog population. Subsequently, a fast SFLA was developed. The key idea was to improve computational speed [22]. Roy Priyanka et al. [23] presented a novel SFLA combined with the crossover of genetic algorithm to avoid falling into local optimum. To enhance the SFLA’s stability and the searching ability, Taher Niknam et al. [24] proposed a chaotic local search algorithm, while Anis Ladgham et al. [25] used an improved SFLA to complete fast MR image segmentation. S. Safaei Arshi et al. [26] proposed multi-objective SFLA for in-core fuel management optimization. Moreover, a multi-phase modified SFLA with external optimization is introduced to solve a multi-depot vehicle routing problem (MDVRP) more quickly [27]. Although their results were satisfactory, there are still many improvements in SFLA, such as dividing the frog population more legitimately, improving local search process, finding a method to update the local worst frog, exchanging global information better, and so forth. Currently, one of the most important improved methods is to combine the SFLA with quantum-inspired theory, which can increase the population diversity, enhance the searching ability, accelerate the convergence rate, and avoid premature. The key idea of the quantum-inspired algorithm is to improve SFLA by a model from quantum computing [28].

The frog population is encoded by the quantum bits in QSFLA. In the processes of local and global search, the frog population is updated by the rotation angle which changes according to local optimum and global optimum. However, there are few researches on QSFLA, and there is no application on sonar image detection according to the authors’ knowledge. Hongyuan Gao et al. [29] introduced a QSFLA and its application in cognitive radio. Later, Lianguo Wang et al. [30] proposed a quantum binary SFLA to solve the 0–1 knapsack problems successfully. The quantum rotation gate is replaced by the phase angle to update the frog individual, which is relatively simple and can avoid premature. To enhance the searching ability, Weiping Ding et al. [31, 32] proposed a minimum attribute self-adaptive cooperative co-evolutionary reduction algorithm based on quantum elitist frogs, in which all frog individuals in a sub-population participate in the local search. However, this algorithm has very high time complexity, and it is also difficult to determine the quantum rotation angle. Sonar images have amounts of noise and relatively weak contrast. Although some noise can be removed by the proposed denoising method in this paper, it is still difficult to obtain relatively accurate detection results by QSFLA [31, 32]. For solving the problem that the QSFLA cannot precisely and quickly detect underwater objects in sonar image because of search mechanism, QSFLA-NSM is given in this paper. Using the clustering centers to describe the frog population after the proposed denoising method in this paper, each frog individual can be directly encoded by real numbers to greatly simplify the evolution process of QSFLA. Meanwhile, to evaluate the frog positions more accurately, a fitness function combining intra-class difference with inter-class difference is adopted. In the process of search, a new search mechanism that combines the quantum search mechanism of QPSO with SFLA is proposed to improve the searching ability and detection accuracy. Simultaneously, the time complexity can also be further reduced. Specifically, the local attractor point is obtained by the local best frog in the sub-population and the global best frog in the whole population. Moreover, the new search mechanism does not need to calculate the average best position of the whole population to update the frog individual, on the contrary, it utilizes the local best frog to update the worst frog in the sub-population. If the worst frog in the sub-population is not improved, the local best frog is replaced by the global best frog in the whole population, and the worst frog in the sub-population is updated again. If the worst frog in the sub-population still is not improved, the new frog individual is randomly generated to replace the worst frog in the sub-population.

The proposed underwater sonar image detection method is applied to original sonar images, the simulation results show the good effectiveness and a powerful searching ability. Compared with the results of QSFLA, SFLA, QPSO, PSO, the quantum genetic algorithm (QGA) and GA, the better adaptability of the proposed method is further demonstrated by the UCI data sets and the benchmark functions. The proposed method has important theoretical and practical value.

## Non-local spatial information denoising method with a novel filtering degree parameter

### Non-local spatial information

**Fig 1:**
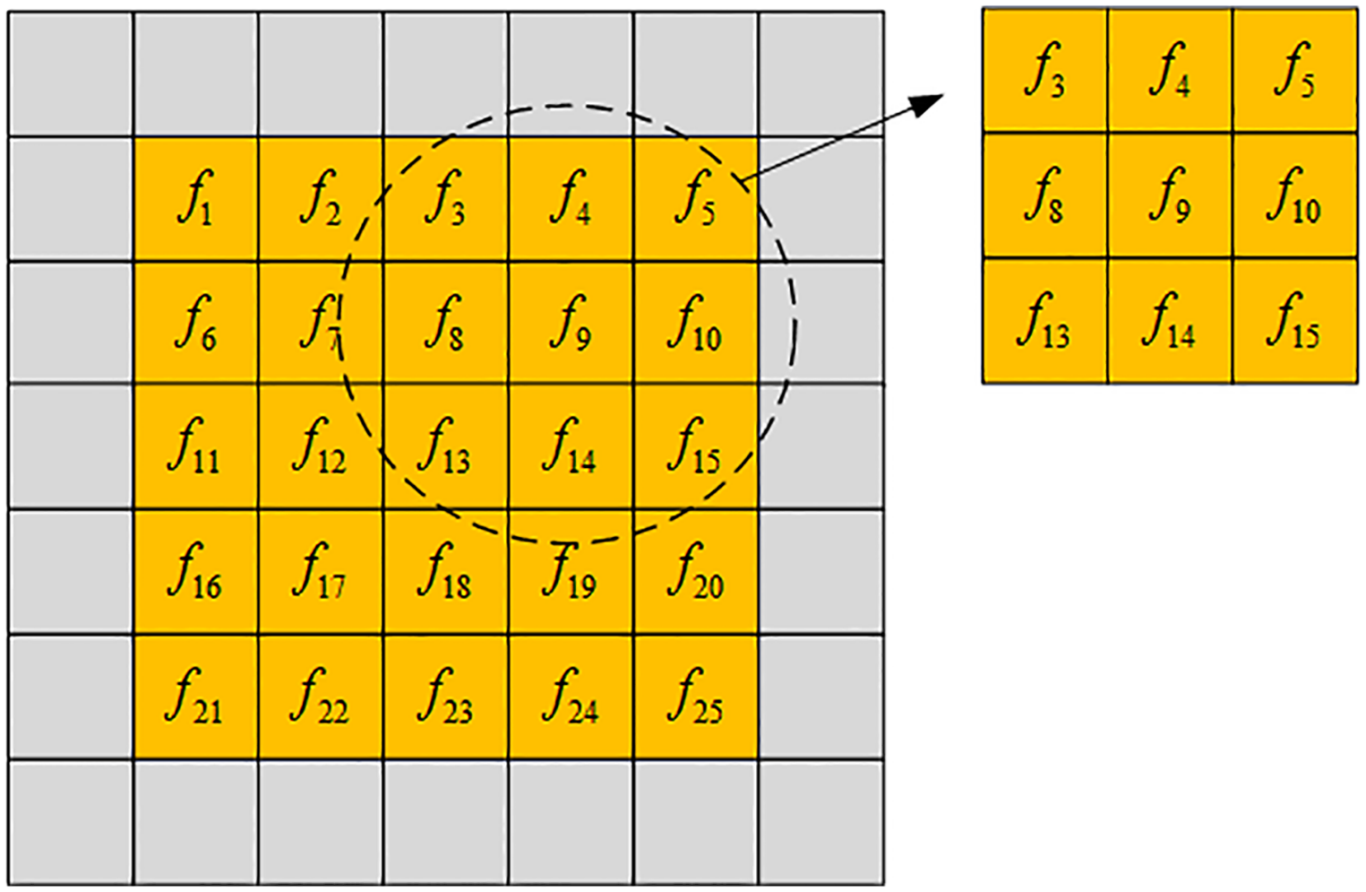
Schematic diagram of search window and neighborhood window.

The search window and the neighborhood window are defined in non-local spatial information. Suppose the search window radius is 2, and the neighborhood window radius is 1. The schematic diagram is shown in Fig 1.

As depicted in Fig 1, {*f*_1_,*f*_2_,⋯,*f*_25_} is a search window, and *f*_13_ is the center pixel of the search window. There is a neighborhood window for every pixel in the search window. For example, {*f*_3_,*f*_4_,*f*_5_,*f*_8_,*f*_9_,*f*_10_,*f*_13_,*f*_14_,*f*_15_} is the neighborhood window of *f*_9_.

A sonar image is defined as *X* = {*x*_1_,*x*_2_,⋯,*x*_*n*_}, which contains *n* pixels. *x*_*j*_ is the j-th pixel in sonar image, and its non-local spatial information $\bar{x_{j}}$ is expressed as follows [11]:

$$\bar{x_{j}}=\sum_{p\in W_{j}^{r}}w_{jp}x_{p},$$ (1)

where $W_{j}^{r}$ is the search window, its center is the j-th pixel *x*_*j*_ and the radius is *r*. The weight *w*_*jp*_ represents the similarity of neighborhood configuration between the center pixel *x*_*j*_ and the p-th pixel *x*_*p*_, 0≤*w*_*jp*_≤1, $\sum_{p\in W_{j}^{r}}w_{jp}=1$.

*w*_*jp*_ is expressed as follows:

$$w_{jp}=\frac{1}{Z_{j}}\text{exp}\left(-{\left\|x\left(N_{j}\right)-x\left(N_{p}\right)\right\|}_{2,\rho }^{2}/h\right),$$ (2)

$$Z_{j}=\sum_{p\in W_{j}^{r}}\text{exp}\left(-{\left\|x\left(N_{j}\right)-x\left(N_{p}\right)\right\|}_{2,\rho }^{2}/h\right),$$ (3)

where *N*_*j*_ represents the neighborhood window whose center is the j-th pixel *x*_*j*_ and the radius is *s*. Similarly, *N*_*p*_ represents the neighborhood window whose center is the p-th pixel *x*_*p*_ and the radius is *s*. Then, *x*(*N*_*j*_) is the vector that contains all the pixels in the neighborhood window *N*_*j*_. Meanwhile, *x*(*N*_*p*_) is the vector that contains all the pixels in the neighborhood window *N*_*p*_. The filtering degree parameter *h* controls the decay of the exponential function, and ${\left\|x\left(N_{j}\right)-x\left(N_{p}\right)\right\|}_{2,\rho }^{2}$ is the weighted Euclidean distance.

${\left\|x\left(N_{j}\right)-x\left(N_{p}\right)\right\|}_{2,\rho }^{2}$ is expressed as follows [10]:

$${\left\|x\left(N_{j}\right)-x\left(N_{p}\right)\right\|}_{2,\rho }^{2}=\sum_{q=1}^{{\left(2s+1\right)}^{2}}\rho^{\left(q\right)}{\left(x^{\left(q\right)}\left(N_{j}\right)-x^{\left(q\right)}\left(N_{p}\right)\right)}^{2},$$ (4)

where *x*^(*q*)^(*N*_*j*_) is the pixel in the q-th dimension of the vector *x*(*N*_*j*_).

*ρ*^(*q*)^ is defined as

$$\rho^{\left(q\right)}=\sum_{t=\text{max}\left(d,1\right)}^{s}\frac{1}{{\left(2t+1\right)}^{2}s},$$ (5)

$$d=\text{max}\left(\left|y_{q}-s-1\right|,\left|z_{q}-s-1\right|\right),$$ (6)

Where *y*_*q*_ = mod(*q*,(2*s*+1)), *z*_*q*_ = *floor*(*q*,(2*s* + 1)) + 1. (*y*_*q*_,*z*_*q*_) is the coordinates of neighborhood window in the dimension q-th.

### A novel filtering degree parameter

The filtering degree parameter *h* is a very important parameter. Its value can powerfully impact on the weight of neighborhood configuration in the Eqs (2) and (3). Therefore, the filtering degree parameter *h* is closely related to denoising result in non-local spatial information denoising method. Through an analysis of comparative experiment results, when the filtering degree parameter is selected as the mean value or median value, there are the filtering degree parameter values over a wide range. When the filtering degree parameter is relatively large, detail information is lost in the sonar image, especially boundary information. In contrast, when the filtering degree parameter is relatively small, the noise of sonar image cannot be removed effectively.

**Fig 2:**
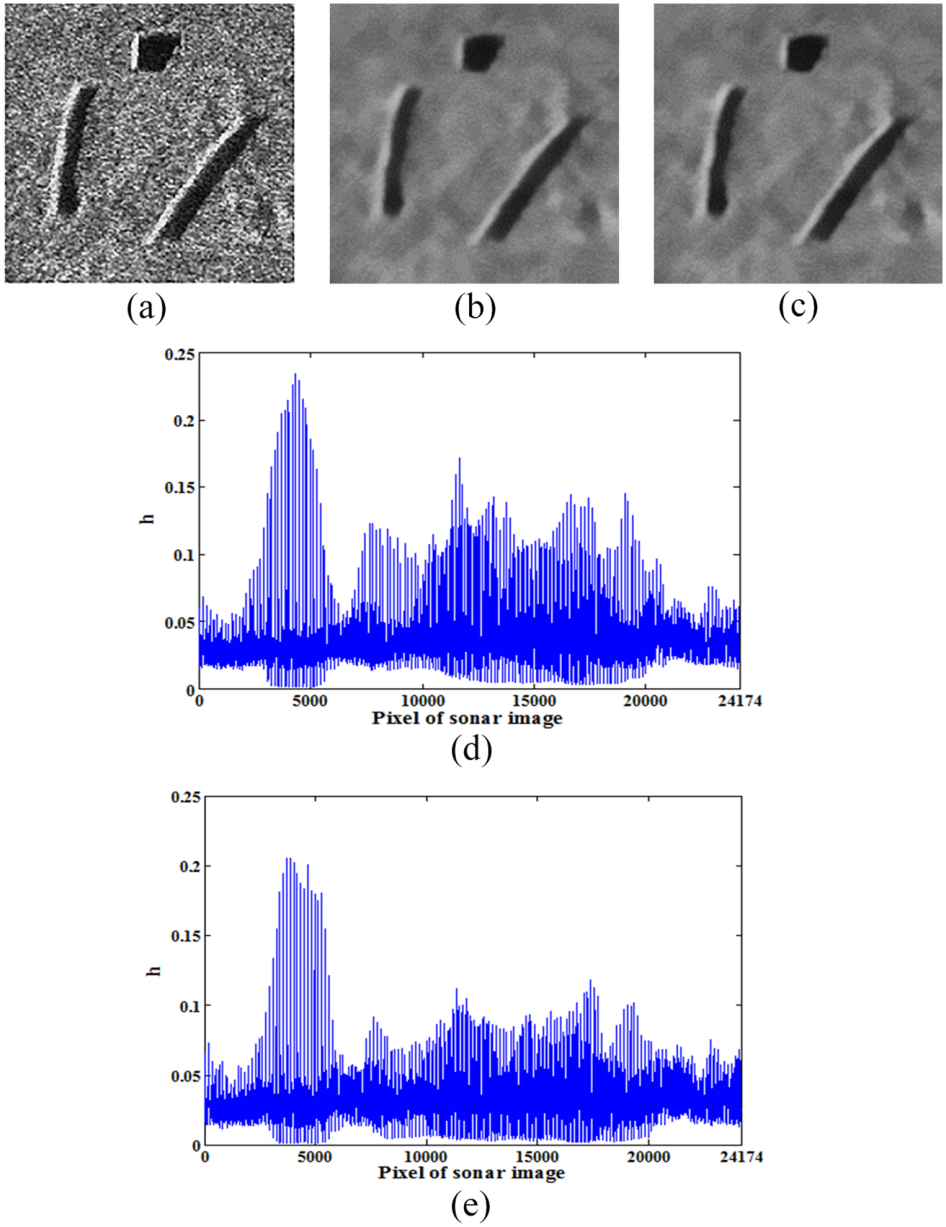
Denoising results of original sonar image using the mean value or the median value (image size: 153×158). (a) original sonar image, (b) and (d) are the denoising result and filtering degree parameter respectively when *h* is the mean value (*r =* 5, *s =* 2), (c) and (e) are the denoising result and filtering degree parameter respectively when *h* is the median value (*r =* 5, *s =* 2).

Fig 2 shows the denoising results of an original sonar image with floating objects when the selected filtering degree parameter is the mean value or the median value.

As can be seen from Fig 2(d) and 2(e), most of the filtering degree parameter values are less than 0.05. But there are still many abnormal parameter values, some of which are greater than 0.1 and some of which are significantly less than 0.01. The abnormally large parameter values lead to a loss of detail information in sonar image, especially boundary information. This result causes some noisy regions to be incorrectly detected as underwater objects. At the same time, to effectively remove the noise, the filtering degree parameter cannot be relatively too small.

**Fig 3:**
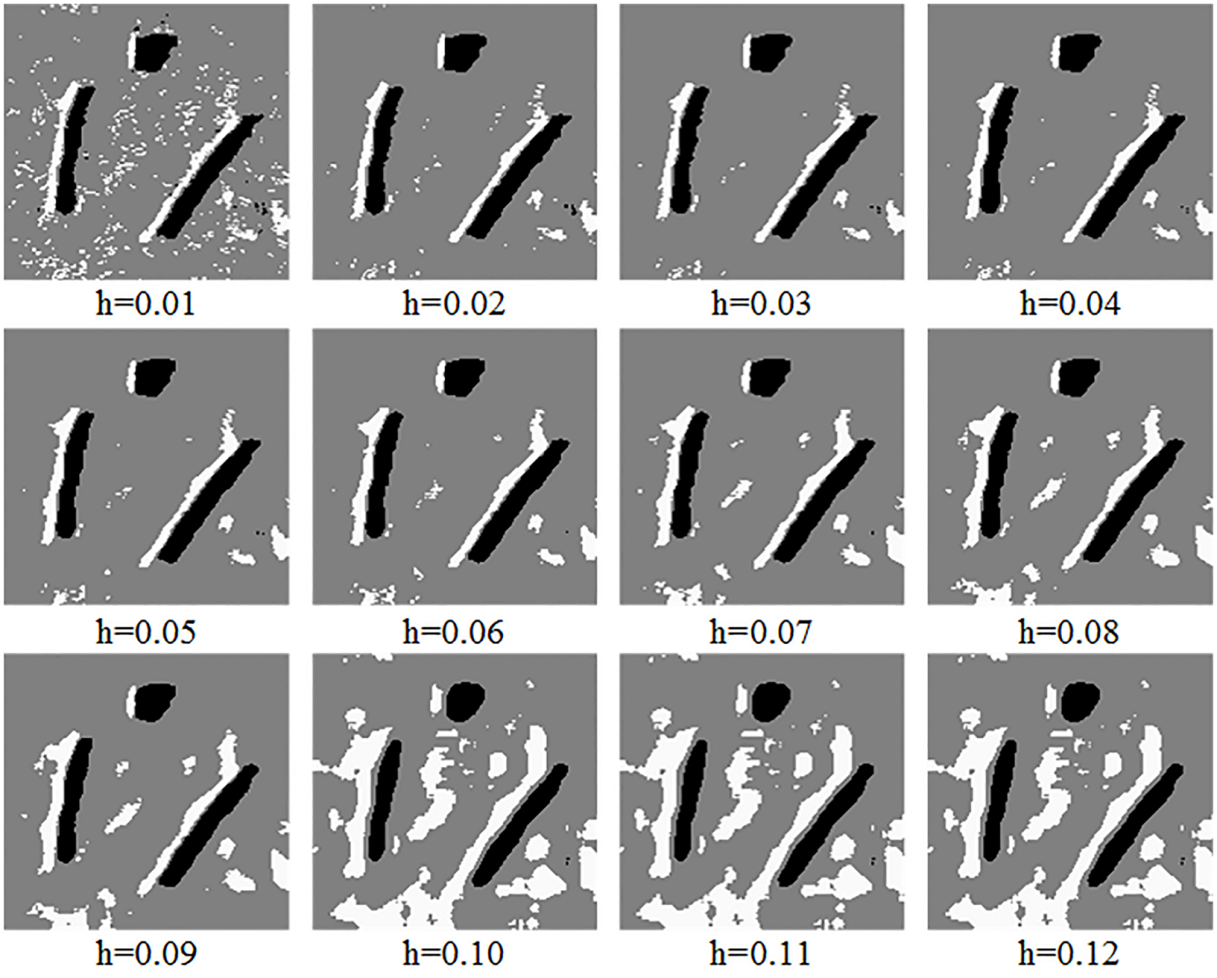
Detection results of FCM: Different filtering degree parameter values.

FCM is a simple, fast and relatively effective algorithm [14]. Therefore, to verify the influence of different filtering degree parameter values on the detection results for sonar image, Fig 3 shows the detection results of FCM on the image shown in Fig 2(a).

As depicted in Fig 3, the large white regions are the object-highlight regions, the large black regions are the shadow regions and the grey region is the background region. The remaining small regions are the isolated regions of detection result, which is caused by the noise. When *h* = 0.01, the detection result is not ideal, and when *h*>0.05, all detection results are getting worse. Therefore, the filtering degree parameter should be neither relatively large nor small. To effectively remove the noise, a novel filtering degree parameter is proposed in this paper. Two threshold values defined as *hmax* and *hmin* respectively are set to remove relatively large and small parameter values. This can improve the denoising performance of non-local spatial information to some extent and is also conducive to the subsequent detection of underwater objects.

For every pixel *x*_*j*_ in sonar image, its filtering degree parameter *h* is defined as

$$hm=\underset{p\in W_{j}^{r}}{\text{mean}}\left\{{\left\|x\left(N_{j}\right)-x\left(N_{p}\right)\right\|}_{2,\rho }^{2}\right\},$$ (7)

$$h=\left\{ \begin{array}{l} hmax\begin{array}{cc} & \begin{array}{cc} & hm>hmax \end{array} \end{array}\\ hm\begin{array}{ccc} & & hmin\leq hm\leq hmax \end{array}\\ hmin\begin{array}{cc} & \begin{array}{cc} & hm<hmin \end{array} \end{array} \end{array}\right.,$$ (8)

where *hmax* = 0.05 and *hmin* = 0.01 according to characteristics of sonar image.

**Fig 4:**
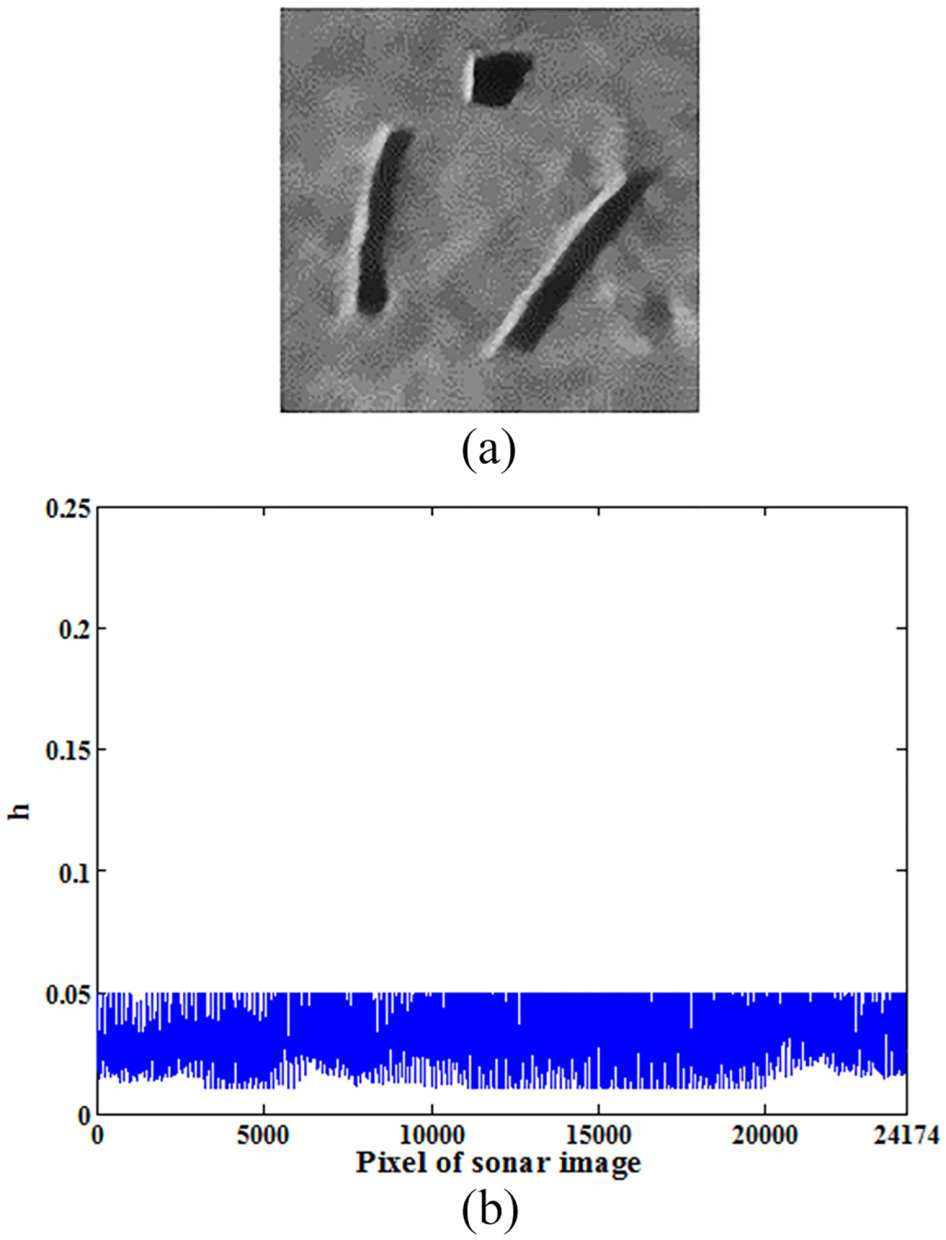
Denoising results of original sonar image with a novel filtering degree parameter (image size: 153×158). (a) denoising result of the proposed denoising method in this paper, (b) filtering degree parameter when *h* is selected according to Eq (8).

Fig 4 shows the denoising results of non-local spatial information with this novel filtering degree parameter on the image shown in Fig 2(a).

As shown in comparative results, the proposed novel filtering degree parameter is relatively stable, and its values are mainly distributed in the interval [0.01, 0.05].

### Effective evaluation of the proposed denoising method

The normalized mean squared error (NMSE) [33], the quality factor (QF) [34] and the mean structural similarity (MSSIM) [35] are used to objectively and quantitatively evaluate the denoising result of sonar image in this paper.

Table 1 shows the evaluation analysis results for Figs 2(b), 2(c) and 4(a).

**Table 1:** Objective and quantitative evaluation values.

Figure	h	NMSE	QF	MSSIM
Fig 2(b)	mean value	0.0572	0.6873	0.2795
Fig 2(c)	median value	0.0557	0.7034	0.2968
Fig 4(a)	novel value	0.0533	0.7162	0.3055

As listed in Table 1, NMSE is the smallest, QF and MSSIM are the largest in Fig 4(a). Therefore, the proposed denoising method can obtain better denoising result of sonar image in this paper.

**Fig 5:**
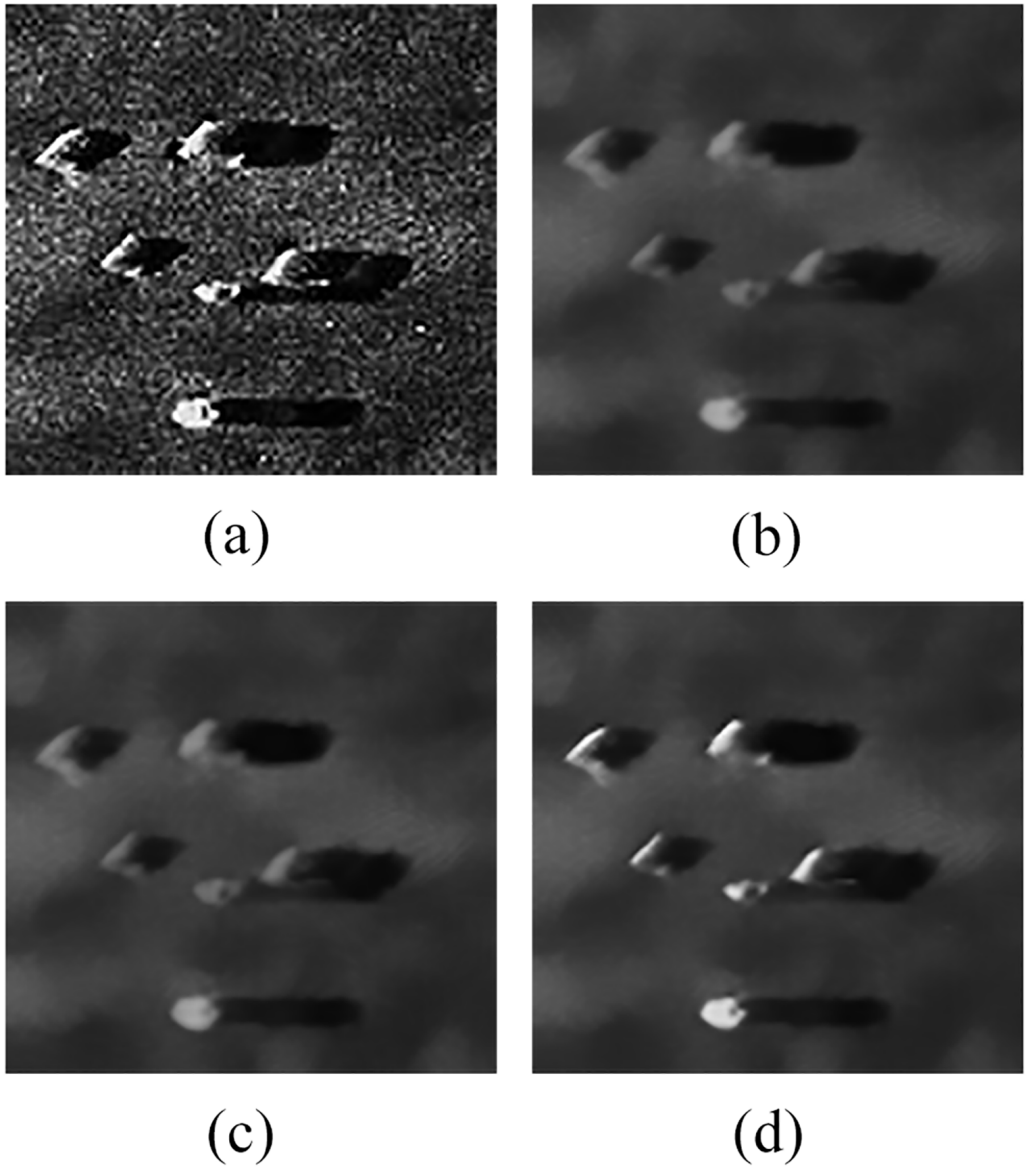
Denoising results of original sonar image (image size: 112×117). (a) original sonar image, (b) denoising result when *h* is the mean value (*r =* 5, *s =* 2), (c) denoising result when *h* is the median value (*r =* 5, *s =* 2), (d) denoising result of the proposed denoising method in this paper.

To verify the adaptability of the proposed denoising method, Fig 5 shows the denoising results of the original sonar image with underwater stones on the bottom, which has relatively weak contrast.

Table 2 shows the evaluation analysis results for Fig 5(b), 5(c) and 5(d).

**Table 2:** Objective and quantitative evaluation values in Fig 5.

Figure	h	NMSE	QF	MSSIM
Fig 5(b)	mean value	0.1253	0.6856	0.2441
Fig 5(c)	median value	0.1251	0.6846	0.2531
Fig 5(d)	novel value	0.1001	0.7741	0.2702

**Fig 6:**
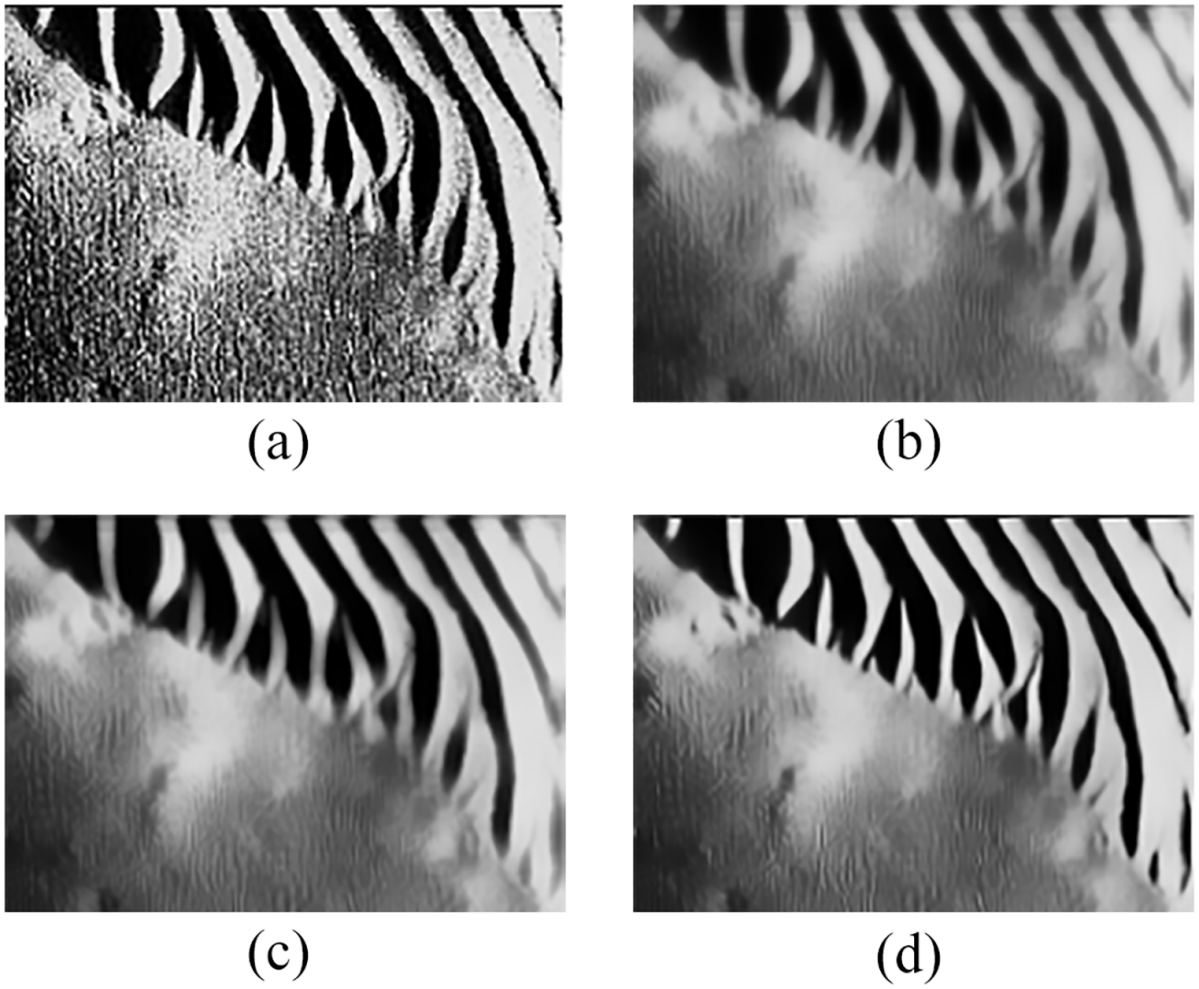
Denoising results of original sonar image (image size: 259×368). (a) original sonar image, (b) denoising result when *h* is the mean value (*r =* 5, *s =* 2), (c) denoising result when *h* is the median value (*r =* 5, *s =* 2), (d) denoising result of the proposed denoising method in this paper.

Fig 6 shows the denoising results of structured seabed, which is an object in sand ripples.

Table 3 shows the evaluation analysis results for Fig 6(b), 6(c) and 6(d).

**Table 3:** Objective and quantitative evaluation values in Fig 6.

Figure	h	NMSE	QF	MSSIM
Fig 6(b)	mean value	0.0622	0.9076	0.4851
Fig 6(c)	median value	0.0582	0.9202	0.5275
Fig 6(d)	novel value	0.0334	0.9549	0.5882

The following conclusions can be drawn by the preceding comparative experiments, NMSE is the smallest, QF and MSSIM are the largest in Figs 5(d) and 6(d). Therefore, the proposed denoising method can avoid the disadvantages of filtering degree parameter in non-local spatial information, which can effectively remove noise, and has a certain adaptability. Meanwhile, the object-highlight and shadow regions are more prominent compared with the background region, so these results are conducive to the subsequent detection of underwater objects.

## QSFLA-NSM

In QSFLA, the frog population is usually encoded by the quantum bits and updated by quantum rotation gate. However, the time complexity of this search mechanism is very high, and it is also difficult to determine the quantum rotation angle. Compared with QSFLA, the search mechanism of QPSO needs fewer parameters [36], and each particle can be directly encoded by real numbers to greatly simplify the evolution process. Meanwhile, particles move in delta potential well of the search space [37]. The congregation of the particle swarm doesn’t lose the randomness and particles can appear on any position of the whole space which is searched in a certain probability. When one particle finds an optimal state, the others quickly converge to it [38, 39]. Therefore, QPSO has relatively good searching ability. But many studies showed that the algorithm structure of QPSO and PSO is not as good as that of QSFLA and SFLA. SFLA combines the advantages of Memetic Algorithm (MA) in terms of genetic evolution and PSO algorithm in terms of social behavior, which balances global exploration ability and local development ability [18, 19]. Therefore, a new search mechanism that combines the quantum search mechanism of QPSO with SFLA is proposed to precisely and quickly detect underwater objects in sonar images. Specifically, the local attractor point is obtained by the local best frog in the sub-population and the global best frog in the whole population. Moreover, the new search mechanism does not need to calculate the average best position of the whole population to update the frog individual, on the contrary, it utilizes the local best frog to update the worst frog in the sub-population. If the worst frog in the sub-population is not improved, the local best frog is replaced by the global best frog in the whole population, and the worst frog in the sub-population is updated again. If the worst frog in the sub-population still is not improved, the new frog individual is randomly generated to replace the worst frog in the sub-population.

### Population initialization

In QSFLA-NSM, each frog individual is directly encoded by real numbers, which greatly simplifies the evolution process. Meanwhile, the proposed algorithm is based on a clustering model in this paper, the object-highlight region, shadow region and background region in sonar image are detected by clustering centers. Therefore, the frog population is described by the clustering centers. Assuming that the number of clusters is *k*, the population size is *N*, the whole population can be expressed as follows:

$$M=\left(\begin{array}{ccc} c_{11} & \ldots & c_{1k}\\ \vdots & \ddots & \vdots \\ c_{N1} & \cdots & c_{Nk} \end{array}\right),$$ (9)

where *c*_*ij*_(1≤*i*≤*N*,1≤*j*≤*k*) is the j-th clustering center of the i-th frog individual.

### Fitness function

The fitness function is mainly used to evaluate the frog positions. The frog population is described by the clustering centers which divide the pixels into different classes in sonar image. The pixels with similar characteristics are divided into the same class, while the pixels with different characteristics are divided into different classes. Generally, when the intra-class difference is minimum and the inter-class difference is maximum simultaneously, the clustering method is ideal. Therefore, a fitness function combining intra-class difference with inter-class difference is used in this paper to evaluate the frog positions more accurately [40].

Assuming that *C* = (*c*_1_,*c*_2_,⋯,*c*_*k*_) represents clustering centers and the pixels in sonar image are expressed as *v*, the intra-class difference *U*_*i*_ is given by

$$U_{i}=\frac{1}{N_{i}}\sum_{v\in G_{i}}\left\|v-c_{i}\right\|.$$ (10)

The inter-class difference between the i-th and j-th clustering center is given by

$$Dis\left(i,j\right)=\left\|c_{i}-c_{j}\right\|,$$ (11)

where *G*_*i*_ is the set of pixels belonging to the i-th clustering center, *i* = 1,2,⋯,*k*, *N*_*i*_ represents the cardinality of the set.

According to the Eqs (10) and (11), the maximum ratio of the intra-class difference and inter-class difference is expressed as follows:

$$F_{i}={\text{max}}_{j,j\neq i}\left\{\frac{U_{i}+U_{j}}{Dis\left(i,j\right)}\right\},$$ (12)

The average value of *F*_*i*_(*i* = 1,2,⋯,*k*) is given by

$$EV=\frac{1}{k}\sum_{i=1}^{k}F_{i},$$ (13)

where less *EV* value indicates the better clustering effect.

The fitness function is expressed as follows:

$$fitness=\frac{1}{EV}=\frac{k}{\sum_{i=1}^{k}{\text{max}}_{j,j\neq i}\left\{\frac{U_{i}+U_{j}}{Dis\left(i,j\right)}\right\}}.$$ (14)

### New search mechanism

The initial frog population is generated randomly from the solution space in QSFLA-NSM. Then, the whole population is sorted and divided into several sub-populations. A local search is carried out in every sub-population. Then, global information exchange is completed by mixing all the frog individuals again.

Assuming that the whole population contains *N* frog individuals, the frog population is defined as *M* = [*M*_1_,*M*_2_,⋯,*M*_*N*_], where the i-th frog individual is *M* = [*M*_*i*1_,*M*_*i*2_,⋯,*M*_*iD*_] and *D* is the dimension of a frog individual. According to the fitness function values, the frog population is sorted in descending order. Then, the frog population is divided into *a* sub-populations, in which every sub-population contains *b* frog individuals (*N* = *a*×*b*). The first frog is assigned to the first sub-population, the second frog is assigned to the second sub-population, …, the a-th frog is assigned to the a-th sub-population, the (a+1)-th frog is assigned to the first sub-population, and so on. This process continues until all the frogs have been assigned to the sub-populations.

The local worst frog is updated, which is called the local search in every sub-population. In the searching process, a new search mechanism that combines the quantum search mechanism of QPSO with SFLA is proposed to improve the searching ability and detection accuracy. Meanwhile, the time complexity can also be further reduced.

The local attractor point is obtained by the local best frog in the sub-population and the global best frog in the whole population. For every sub-population, the local worst frog is defined as *M*_*w*_, and the local best frog is defined as *M*_*b*_. The global best frog of whole population is defined as *M*_*g*_. The local attractor point of the sub-population can be expressed as follows:

$$P=\frac{rand_{u1}\times M_{b}+rand_{u2}\times M_{g}}{rand_{u1}+rand_{u2}},$$ (15)

where *rand*_*u*1_ and *rand*_*u*2_ are two random numbers of uniform distribution in the interval [0, 1].

When the local worst frog evolves towards the local best frog in sub-population, a new frog individual is generated, which is defined as *newM*_*w*_. The specific updating formulas are as follows:

$$newM_{w}=P-\beta \times \left|M_{b}-M_{w}\right|\times \text{ln}\left(1/rand_{u3}\right)\begin{array}{cc} & \begin{array}{cc} & rand_{u4}\geq 0.5 \end{array} \end{array},$$ (16)

$$newM_{w}=P+\beta \times \left|M_{b}-M_{w}\right|\times \text{ln}\left(1/rand_{u3}\right)\begin{array}{cc} & \begin{array}{cc} & rand_{u4}<0.5 \end{array} \end{array},$$ (17)

where *rand*_*u*3_ and *rand*_*u*4_ are two random numbers of uniform distribution in the interval [0, 1]. β is the contraction–expansion coefficient, which can control the convergence speed of the algorithm and linearly decreases with the iterative times. The specific expression is

$$\beta =Mmax-\left(Mmax-Mmin\right)\times \frac{t}{t_{max}},$$ (18)

where *t* is the current iterative times. *t*_*max*_ is local maximum number of iterations. *M*_*max*_ and *M*_*min*_ are the upper bound and lower bound of β respectively.

The fitness function value of *newM*_*w*_ is computed and compared with the local worst frog *M*_*w*_. If the fitness function value is larger than *M*_*w*_, *newM*_*w*_ will replace *M*_*w*_. Otherwise, the local best frog individual *M*_*b*_ in Eqs (16) and (17) is replaced by the global best frog *M*_*g*_, and the local worst frog individual *M*_*w*_ is updated again. If there is still no improvement, a new frog individual is randomly generated within the feasible solution space to replace *M*_*w*_. This updating process of local search continues until the local maximum number of iterations is reached.

After the local search, all the frog individuals in the whole population are mixed and sorted again, which is called the global information exchange. Then, the frog population is divided into sub-populations again and the local search in every sub-population is carried out. This cycle continues until the global maximum number of iterations is reached.

## Experimental results and analysis

This section shows numerical examples to validate the generality and effectiveness of the proposed models for sonar image detection. These results were obtained using Matlab 2011B with a 2.5 GHz Pentium processor and 4 GB of RAM. The relevant parameters are as follows: the number of clustering centers is 3, the global maximum number of iterations is 10, the local maximum number of iterations is 5, the population size is 12, and the number of sub-populations is 6.

**Fig 7:**
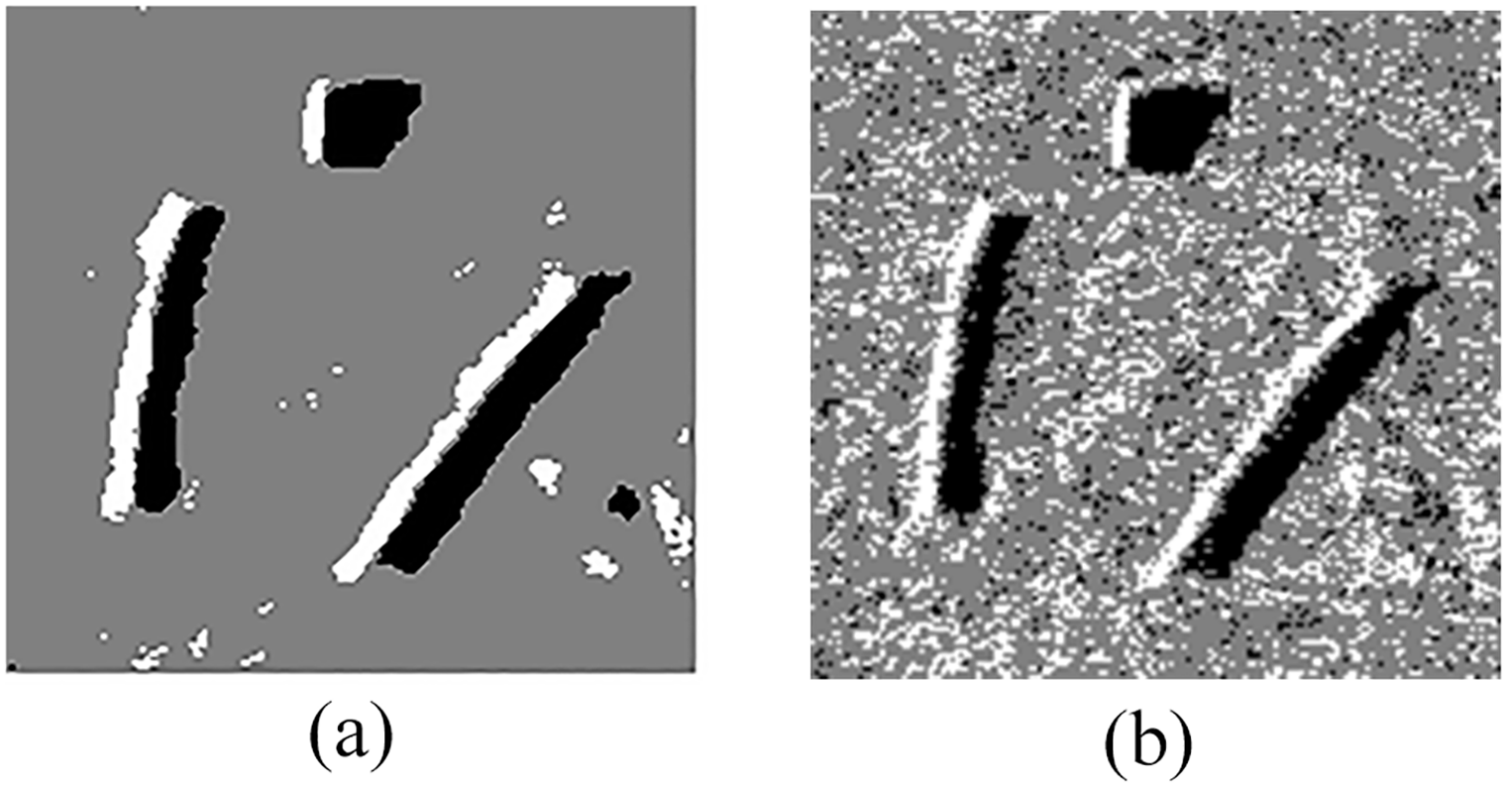
Detection results of original sonar image (image size: 153×158). (a) detection result of QSFLA-NSM on the image shown in Fig 4(a), and (b) detection result of QSFLA-NSM on the image shown in Fig 2(a).

An example of the detection results is shown in Fig 7.

As depicted in Fig 7, the non-local spatial information denoising method with a novel filtering degree parameter demonstrates that it can remove noise effectively in this paper, and the result is conducive to the subsequent detection of underwater objects.

**Fig 8:**
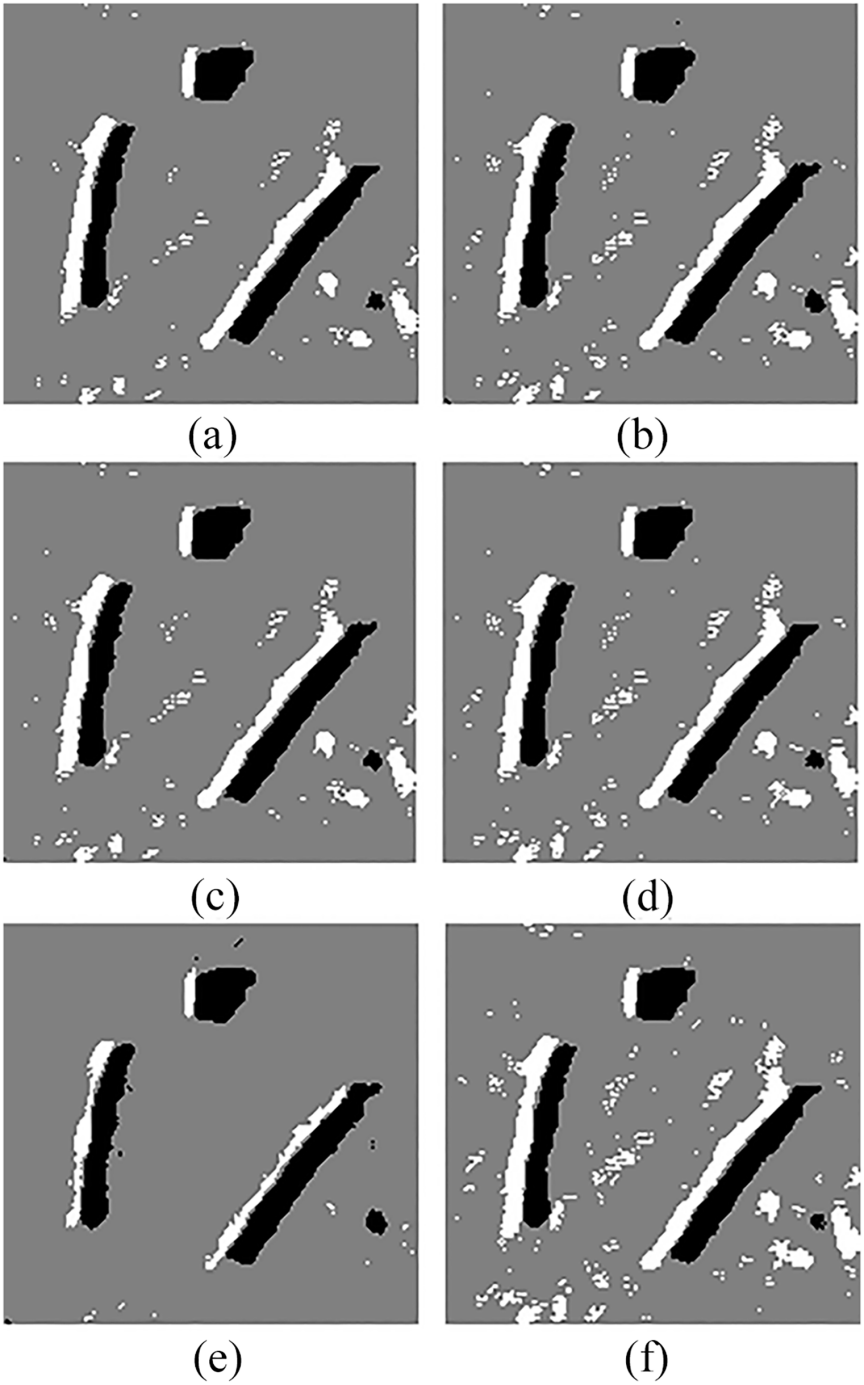
Detection results of original sonar image with other intelligent optimization algorithms (image size: 153×158). (a) detection result of QSFLA, (b) detection result of SFLA, (c) detection result of QPSO, (d) detection result of PSO, (e) detection result of QGA, and (f) detection result of GA.

In order to compare QSFLA-NSM with other intelligent optimization algorithms, Fig 8 shows the comparative results on the denoised image shown in Fig 4(a), which includes QSFLA [31, 32], SFLA [17], QPSO [36], PSO [36], QGA [41], and GA [42].

As shown by the comparison of experimental results, Fig 7(a) contains relatively less noise than Fig 8. Although Fig 8(e) also has less noise, the integrity of the underwater object is seriously weak. Meanwhile, comparing the time complexity of QSFLA-NSM in Fig 7(a) with QSFLA in Fig 8(a), the running time of QSFLA-NSM is 3.71 seconds, while the running time of QSFLA is 6.24 seconds after the same 10 iterations. Therefore, the proposed QSFLA-NSM in this paper also improves on QSFLA with respected to the time complexity.

**Fig 9:**
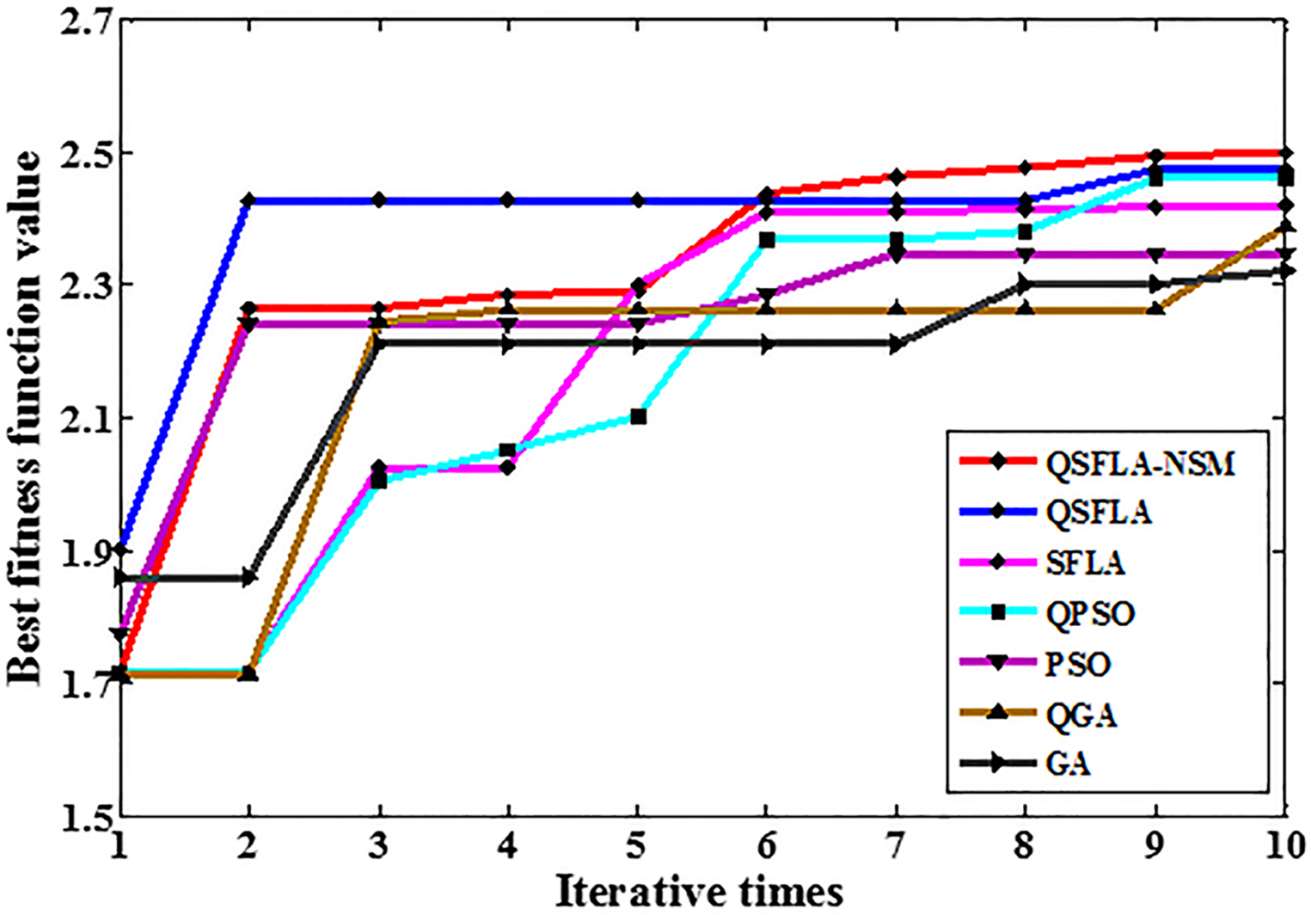
The graph of the best fitness function values in Table 4.

To demonstrate the advantages of QSFLA-NSM more clearly, Table 4 shows the variation of best fitness function values after 10 iterations in Figs 7(a) and 8. To obtain a clearer and more intuitive comparison, Fig 9 shows the graph corresponding to the values listed in Table 4.

**Table 4:** Variation of best fitness function values in Figs 7(a) and 8.

Iterative times	1	2	3	4	5	6	7	8	9	10
QSFLA-NSM	1.717	2.265	2.265	2.287	2.291	2.439	2.463	2.477	2.494	2.500
QSFLA	1.901	2.428	2.428	2.428	2.428	2.428	2.428	2.428	2.474	2.474
SFLA	1.717	1.717	2.025	2.025	2.301	2.409	2.409	2.415	2.417	2.419
QPSO	1.717	1.717	2.004	2.051	2.102	2.369	2.369	2.382	2.461	2.461
PSO	1.774	2.242	2.242	2.242	2.242	2.285	2.347	2.347	2.347	2.347
QGA	1.712	1.712	2.244	2.263	2.263	2.263	2.263	2.263	2.263	2.389
GA	1.860	1.860	2.211	2.211	2.211	2.211	2.211	2.302	2.302	2.321

It can be seen from Table 4 and Fig 9 that the best fitness value of QSFLA-NSM is the largest after 10 iterations, which demonstrates the detection result of QSFLA-NSM is closer to the global optimal solution in this paper.

**Fig 10:**
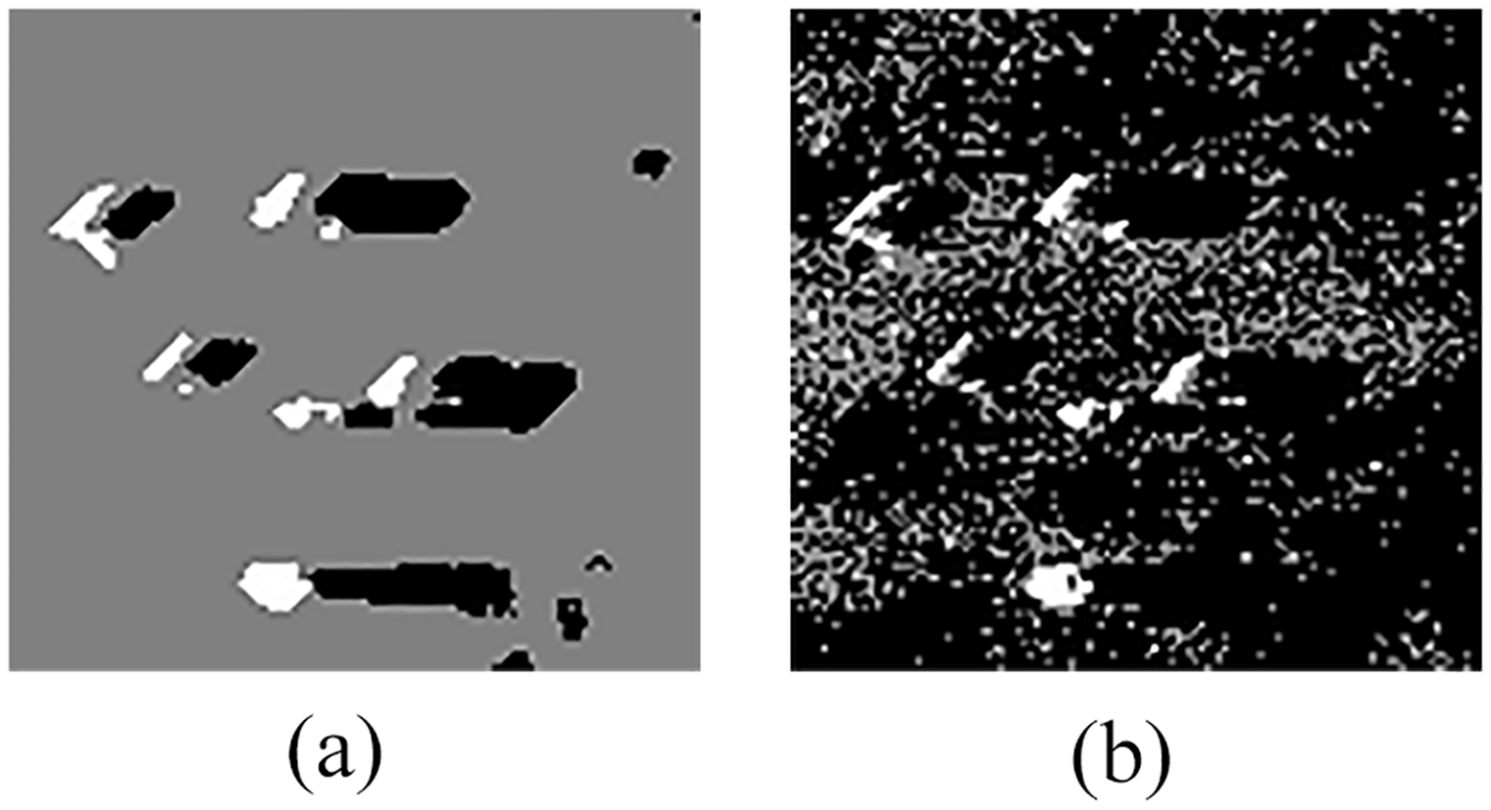
Detection results of original sonar image (image size: 112×117). (a) detection result of QSFLA-NSM on the image shown in Fig 5(d), (b) detection result of QSFLA-NSM on the image shown in Fig 5(a).

**Fig 11:**
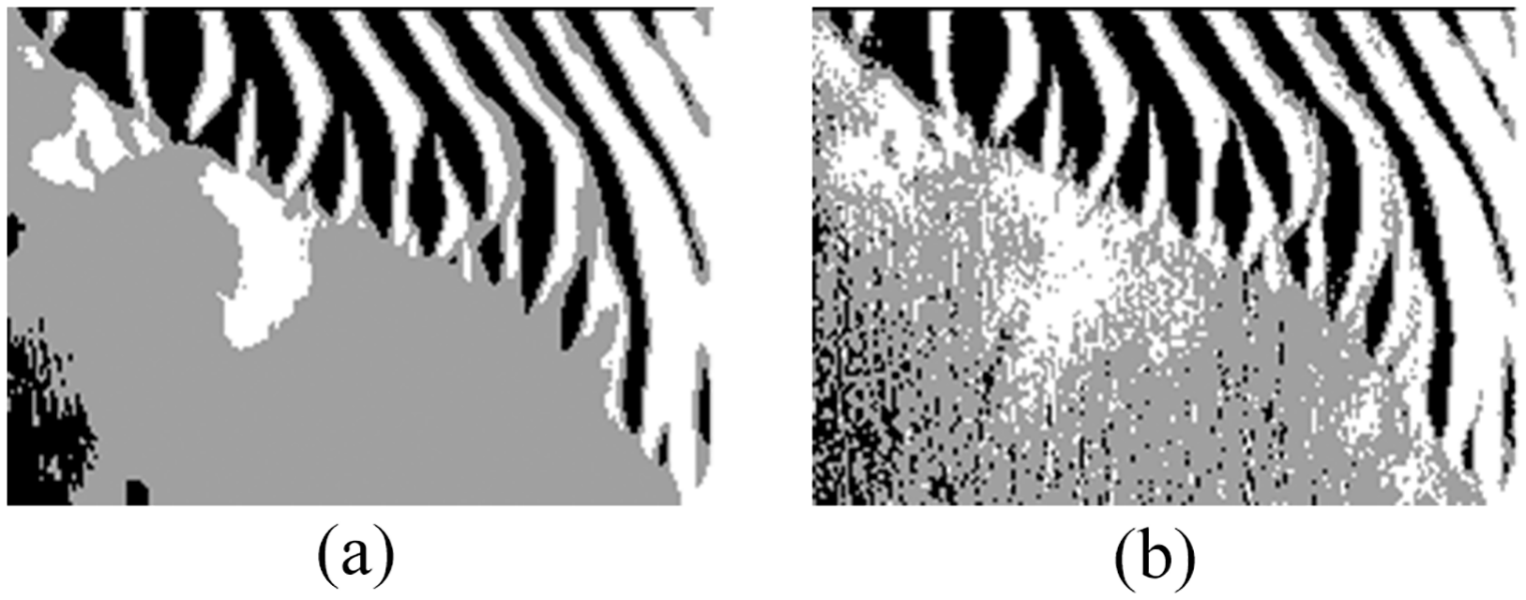
Detection results of original sonar image (image size: 259×368). (a) detection result of QSFLA-NSM on the image shown in Fig 6(d), (b) detection result of QSFLA-NSM on the image shown in Fig 6(a).

Figs 10 and 11 show the detection results on the image shown in Figs 5 and 6 respectively, which further verifies the effectiveness of the proposed models for sonar image detection.

As can be seen from Figs 10 and 11, the comparative experiments further demonstrate that the non-local spatial information denoising method with a novel filtering degree parameter is necessary and plays a vital role in whole detection process.

**Fig 12:**
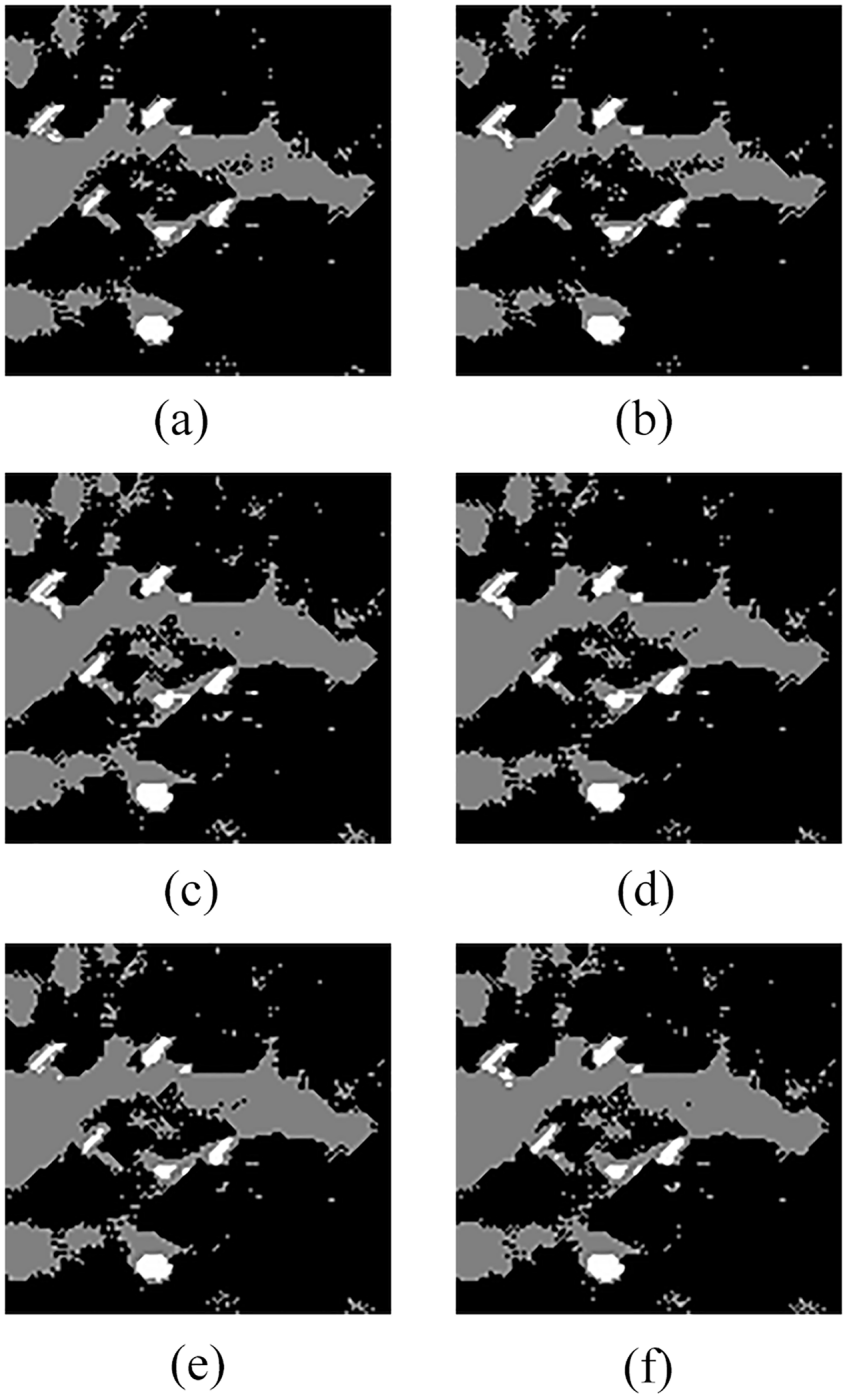
Detection results of original sonar image with other intelligent optimization algorithms (image size: 112×117). (a) detection result of QSFLA, (b) detection result of SFLA, (c) detection result of QPSO, (d) detection result of PSO, (e) detection result of QGA, and (f) detection result of GA.

**Fig 13:**
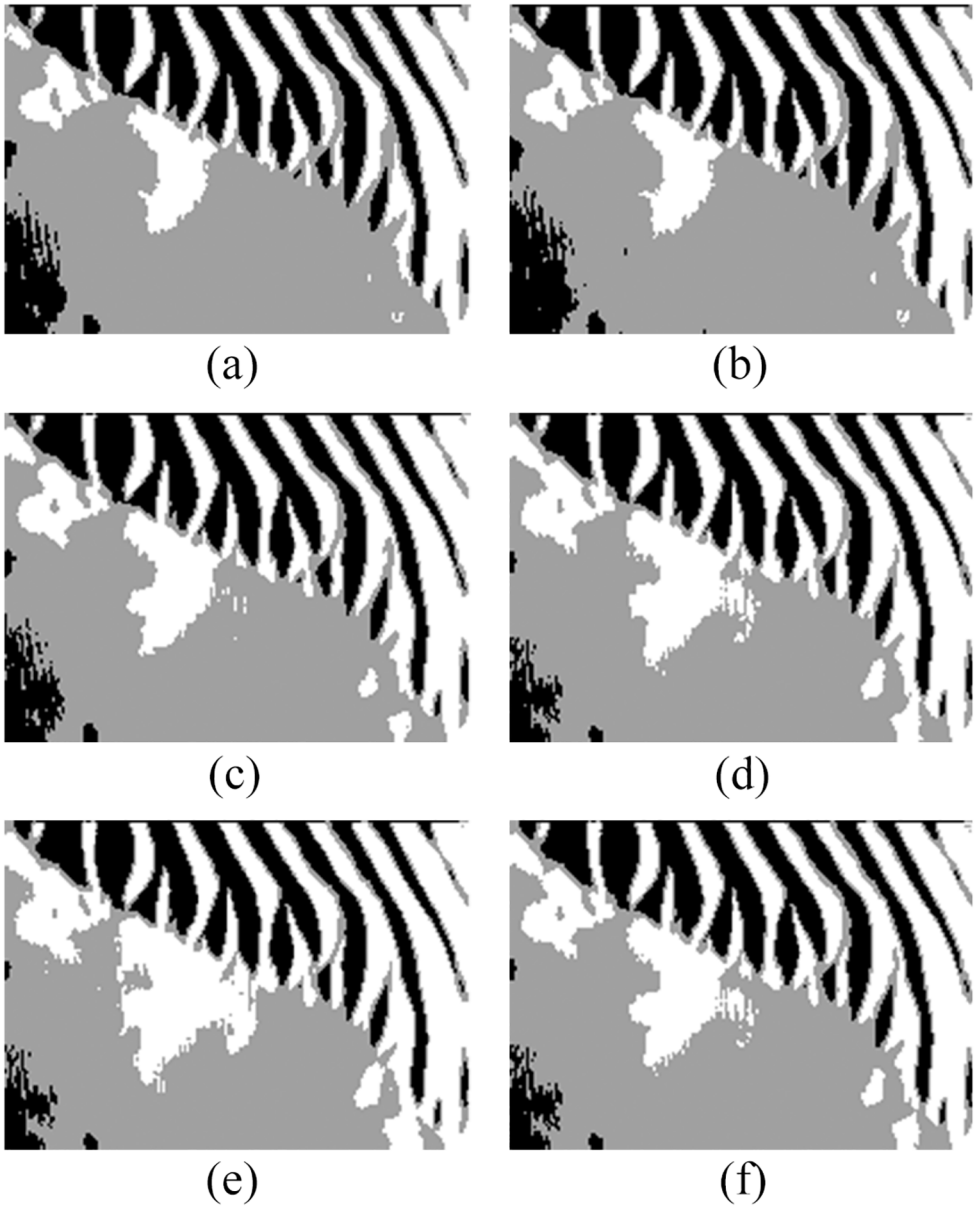
Detection results of original sonar image with other intelligent optimization algorithms (image size: 259×368). (a) detection result of QSFLA, (b) detection result of SFLA, (c) detection result of QPSO, (d) detection result of PSO, (e) detection result of QGA, and (f) detection result of GA.

Similarly, in order to compare QSFLA-NSM with other intelligent optimization algorithms, Figs 12 and 13 show the comparative results on the denoised image shown in Figs 5(d) and 6(d) respectively, which includes QSFLA [31, 32], SFLA [17], QPSO [36], PSO [36], QGA [41], and GA [42].

In contrast to the detection results of other intelligent optimization algorithms, only the proposed QSFLA-NSM can detect the object high-light region and the shadow region better and basically eliminate the influence of noise. Meanwhile, comparing the time complexity of QSFLA-NSM in Figs 10(a) and 11(a) with that of QSFLA in Figs 12(a) and 13(a) respectively, the running time of QSFLA-NSM is 2.07 and 14.949 seconds respectively, while the running time of QSFLA is 3.39 and 23.715 seconds after the same 10 iterations. Therefore, the proposed QSFLA-NSM has lower time complexity in this paper.

**Fig 14:**
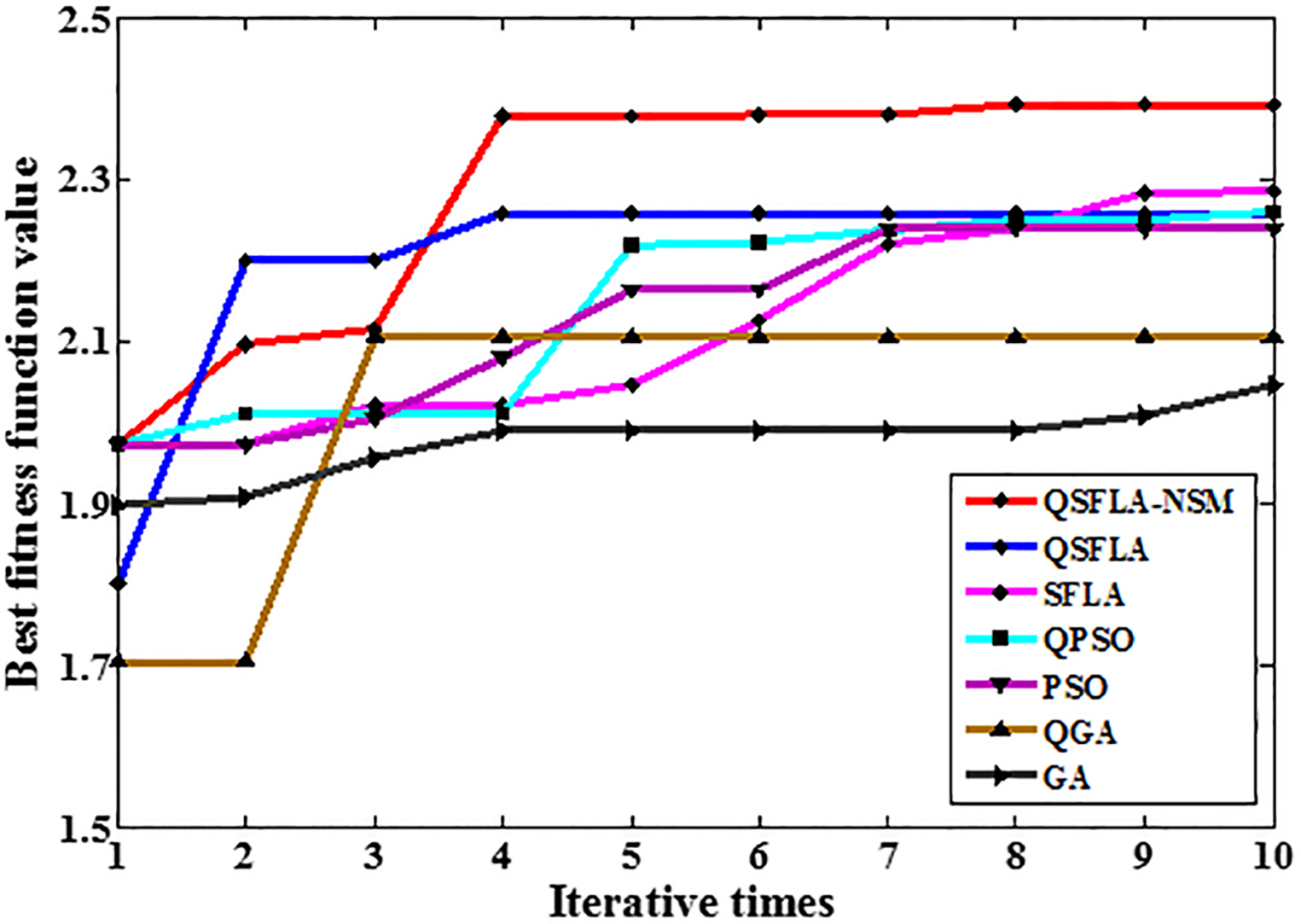
The graph of the best fitness function values in Table 5.

**Fig 15:**
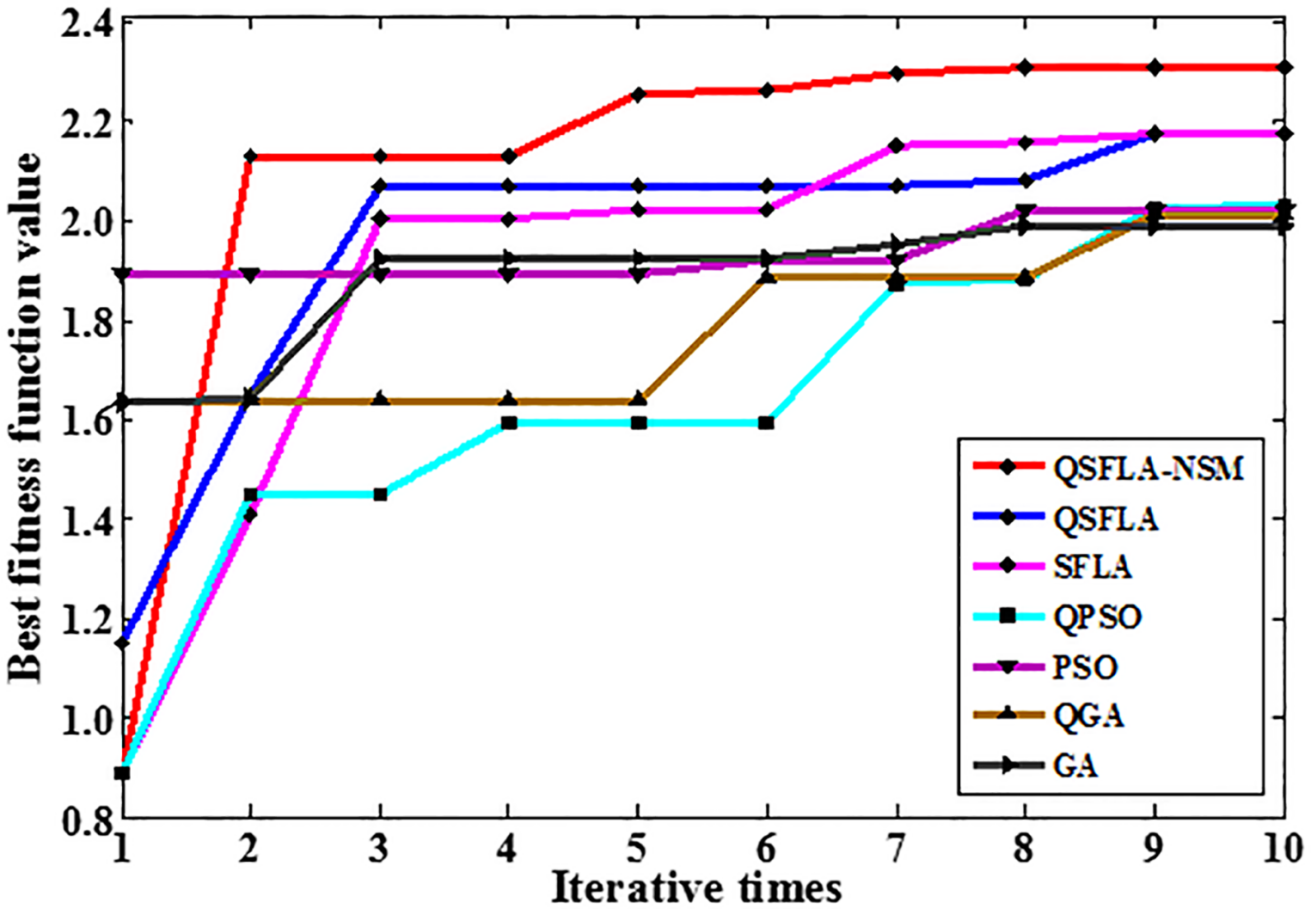
The graph of the best fitness function values in Table 6.

To demonstrate the advantages of QSFLA-NSM more clearly again, Tables 5 and 6 show the variation of best fitness function values after 10 iterations for QSFLA-NSM and other intelligent optimization algorithms. To obtain a clearer and more intuitive comparison, Fig 14 shows the graph of the values listed in Table 5, and Fig 15 shows the graph of the values listed in Table 6.

**Table 5:** Variation of best fitness function value in Figs 10(a) and 12.

Iterative times	1	2	3	4	5	6	7	8	9	10
QSFLA-NSM	1.972	2.097	2.115	2.377	2.377	2.379	2.379	2.391	2.391	2.391
QSFLA	1.800	2.199	2.199	2.258	2.258	2.258	2.258	2.258	2.258	2.258
SFLA	1.972	1.972	2.022	2.022	2.046	2.126	2.220	2.238	2.284	2.285
QPSO	1.972	2.010	2.010	2.010	2.218	2.223	2.236	2.250	2.250	2.259
PSO	1.972	1.972	2.004	2.079	2.163	2.163	2.240	2.240	2.240	2.240
QGA	1.704	1.704	2.105	2.105	2.105	2.105	2.105	2.105	2.105	2.105
GA	1.897	1.908	1.956	1.989	1.989	1.989	1.989	1.989	2.009	2.047

**Table 6:** Variation of best fitness function value in Figs 11(a) and 13.

Iterative times	1	2	3	4	5	6	7	8	9	10
QSFLA-NSM	0.889	2.130	2.130	2.130	2.255	2.263	2.295	2.307	2.307	2.308
QSFLA	1.151	1.648	2.070	2.070	2.070	2.070	2.070	2.080	2.177	2.177
SFLA	0.889	1.4078	2.0052	2.005	2.023	2.023	2.152	2.159	2.175	2.175
QPSO	0.889	1.450	1.4504	1.594	1.594	1.594	1.876	1.881	2.026	2.033
PSO	1.891	1.891	1.8910	1.891	1.891	1.922	1.922	2.021	2.021	2.021
QGA	1.639	1.639	1.639	1.639	1.639	1.887	1.887	1.887	2.011	2.011
GA	1.633	1.642	1.925	1.925	1.925	1.925	1.951	1.999	1.999	1.999

As can be seen from Tables 5 and 6, Figs 14 and 15, the best fitness value of QSFLA-NSM is also the largest after 10 iterations. Therefore, the proposed QSFLA-NSM has a certain effectiveness and adaptability.

**Fig 16:**
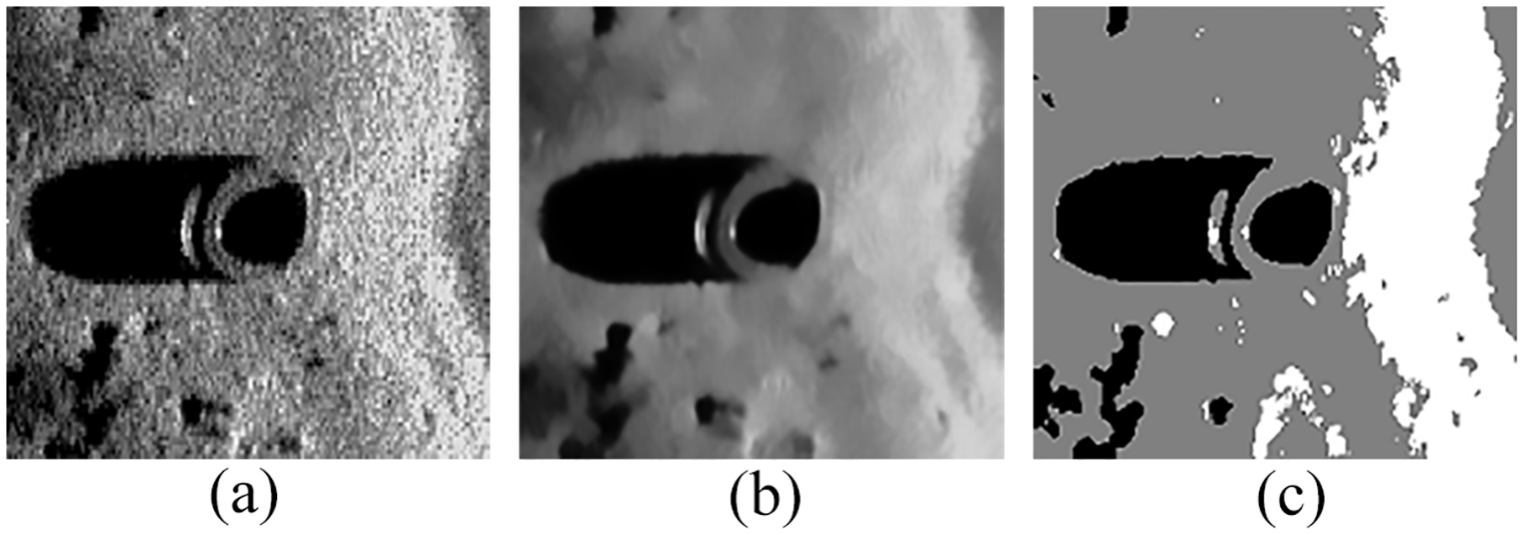
Detection results of original sonar image (image size: 197×211). (a) original sonar image, (b) denoising result of the proposed denoising method in this paper, and (c) detection result of QSFLA-NSM in this paper.

**Fig 17:**
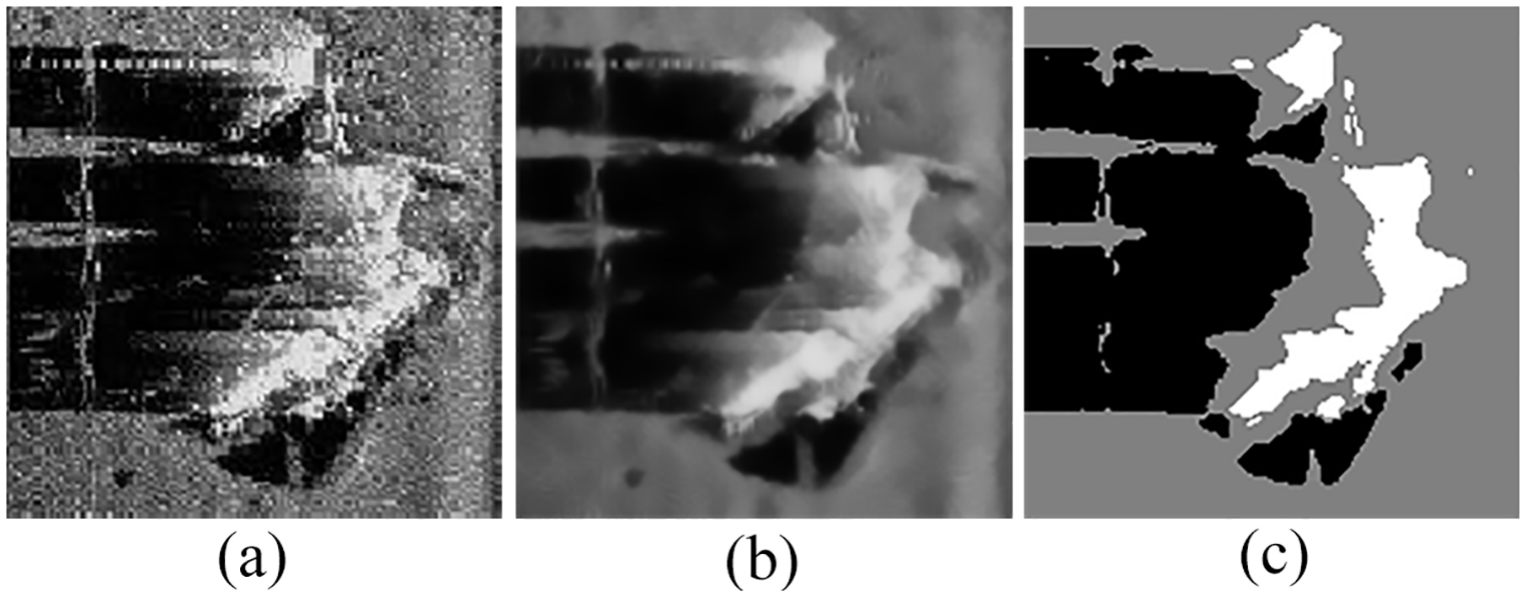
Detection results of original sonar image (image size: 173×167). (a) original sonar image, (b) denoising result of the proposed denoising method in this paper, and (c) detection result of QSFLA-NSM in this paper.

**Fig 18:**
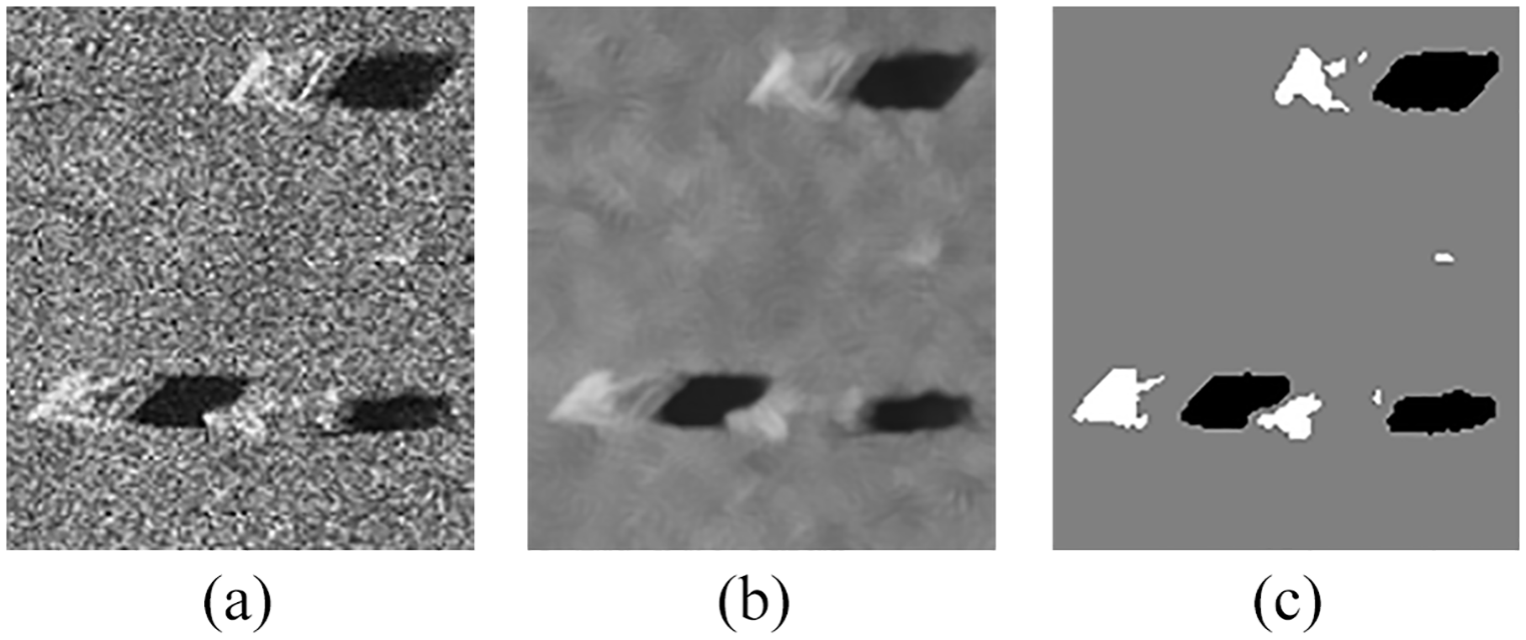
Detection results of original sonar image (image size: 239×205). (a) original sonar image, (b) denoising result of the proposed denoising method in this paper, and (c) detection result of QSFLA-NSM in this paper.

To further verify the adaptability of the proposed denoising method and QSFLA-NSM in this paper, Fig 16 shows the detection results of an original sonar image with underwater tire on the bottom. Fig 17 shows the detection results of larboard original sonar image including rocks, which are buried partly in the sand. Fig 18 shows the detection results of another original sonar image with floating objects.

From the results shown in Figs 16(b), 17(b) and 18(b), the non-local spatial information denoising method with a novel filtering degree parameter can remove noise effectively. It is adaptable for complex underwater sonar images to some extent. The proposed QSFLA-NSM in this paper can detect underwater objects with more accuracy in Figs 16(c), 17(c) and 18(c), and the object-highlight and shadow boundary contours are successfully extracted and detected. The proposed model has a certain effectiveness and adaptability, which can detect original sonar image with floating objects, partly buried objects and objects on the bottom as shown in Figs 16–18. Moreover, it provides better preconditions for the subsequent feature extraction and underwater object recognition.

The proposed QSFLA-NSM is based on clustering model, and therefore the clustering experiments of UCI data sets are carried out to verify the adaptability of QSFLA-NSM, including Iris, Heart, Glass, Breast and Sonar [43]. The dimension, quantity of data and number of classification are shown in Table 7.

**Table 7:** UCI data sets.

UCI	Dimension	Quantity of data	Number of classification
Iris	4	150	3
Heart	13	270	2
Glass	9	214	6
Breast	9	699	2
Sonar	60	208	2

Clustering correct rate is generally used to measure the clustering result. It is the ratio of the number of correctly-clustered samples to the total number. Table 8 shows the clustering correct rates of the proposed QSFLA-NSM and other intelligent optimization algorithms including QSFLA, SFLA, QPSO, PSO, QGA, and GA.

**Table 8:** Clustering correct rates of UCI data sets.

UCI	QSFLA-NSM	QSFLA	SFLA	QPSO	PSO	QGA	GA
Iris	0.9200	0.9067	0.8400	0.8467	0.7600	0.7067	0.6867
Heart	0.6148	0.5963	0.5852	0.5963	0.5815	0.5519	0.5852
Glass	0.4579	0.4439	0.3645	0.3832	0.3178	0.3738	0.3551
Breast	0.9628	0.9642	0.9027	0.9485	0.9399	0.8870	0.9056
Sonar	0.5673	0.5385	0.4856	0.5192	0.5144	0.5192	0.4952

As shown in Table 8, the clustering correct rates of QSFLA-NSM are higher than those of the other intelligent optimization algorithms in the experiments of Iris, Heart, Glass and Sonar. For Breast, the clustering correct rate of QSFLA-NSM is slightly lower than that of QSFLA, but obviously higher than the other five intelligent optimization algorithms.

Table 9 shows the time complexity (s) of different intelligent optimization algorithms.

**Table 9:** Time complexity of different intelligent optimization algorithms (s).

UCI	QSFLA-NSM	QSFLA	SFLA	QPSO	PSO	QGA	GA
Iris	8.34	32.36	6.32	3.47	3.85	4.03	3.42
Heart	10.08	45.15	7.00	4.14	4.11	4.48	4.40
Glass	19.95	89.95	12.96	6.96	7.03	8.00	6.98
Breast	21.85	79.20	16.03	8.27	8.16	8.65	8.51
Sonar	10.03	91.88	6.82	4.31	4.13	5.96	4.07

As can be seen from Table 9, the time complexity of QSFLA is obviously the highest among all the algorithms. The proposed QSFLA-NSM in this paper can reduce the time complexity to some extent.

Similarly, to further verify the searching ability of QSFLA-NSM, four benchmark functions are used to test its searching ability. Griewank, Rastrigrin and Ackley are multimodal and contain many local optima, but only one global optimum. Rosenbrock is unimodal and contains only one global optimum. The function name, search space, optimum value and modality of the selected benchmark functions are listed in Table 10.

**Table 10:** Information of the benchmark functions.

Function	Search space	Optimum value	Modality
Griewank	[−600,600]	0	multimodal
Rastrigrin	[−5.12,5.12]	0	multimodal
Ackley	[−32.768,32.768]	0	multimodal
Rosenbrock	[−2.048,2.048]	0	unimodal

**Fig 19:**
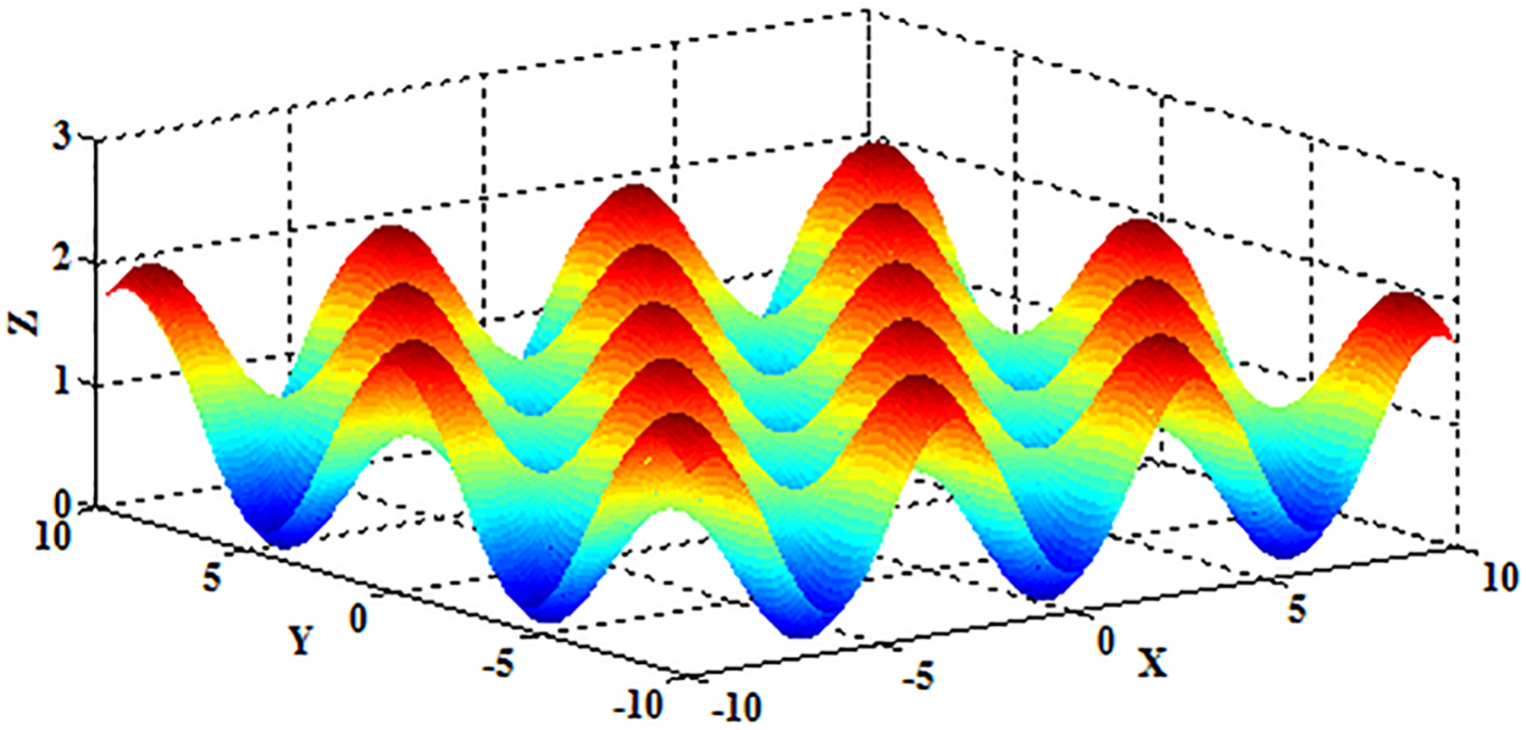
The schematic diagram of the Griewank function.

The Griewank function has many local minima that are regularly distributed. It is a continuous, multimodal, scalable, convex, and quadratic test function [44] and can be represented by

$$f_{1}(x)=\sum_{i=1}^{D}(\frac{x_{i}{}^{2}}{4000})-\prod_{i=1}^{D}\text{cos}(\frac{x_{i}}{\sqrt{i}})+1,$$ (19)

where *x*_*i*_∈[–600,600], and *D* represents the dimension of the independent variable *x*. The schematic diagram is shown in Fig 19 when *D* = 2.

Obviously, the Griewank function is multimodal, and the optimum value 0 can be obtained when (*x*_1_,*x*_2_,⋯,*x*_*D*_) = (0,0,⋯,0).

**Fig 20:**
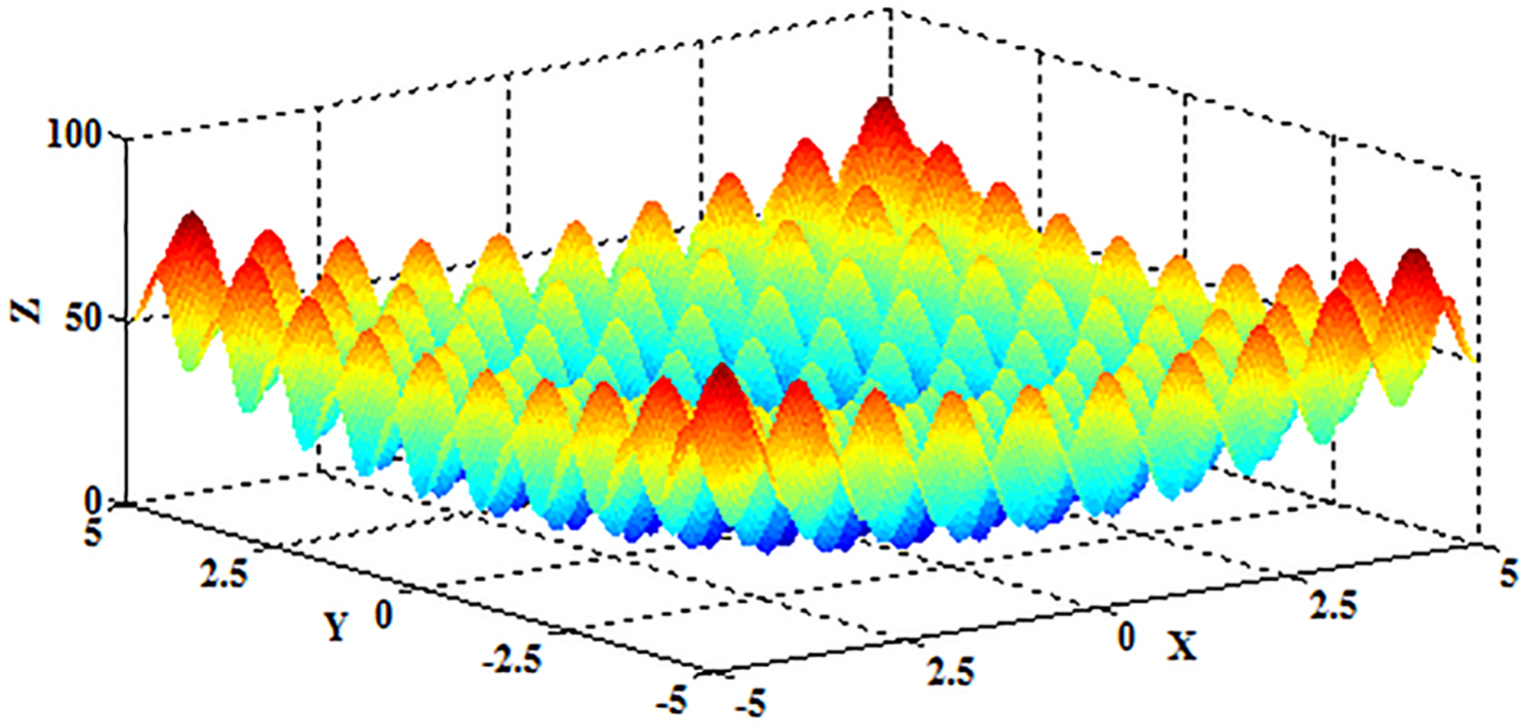
The schematic diagram of the Rastrigrin function.

The Rastrigrin function is a fairly difficult problem due to the large search space and large number of local optima. The function is multimodal and nonlinear, therefore the locations of the optima are regularly distributed [44]. It is given by

$$f_{2}(x)=\sum_{i=1}^{D}\left[x_{i}{}^{2}-10\text{cos}\left(2\pi x_{i}\right)+10\right],$$ (20)

where *x*_*i*_∈[−5.12,5.12], and *D* represents the dimension of the independent variable *x*. The schematic diagram is shown in Fig 20 when *D* = 2.

As depicted in Fig 20, the Rastrigrin function is multimodal, and the optimum value 0 can be obtained when (*x*_1_,*x*_2_,⋯,*x*_*D*_) = (0,0,⋯,0).

**Fig 21:**
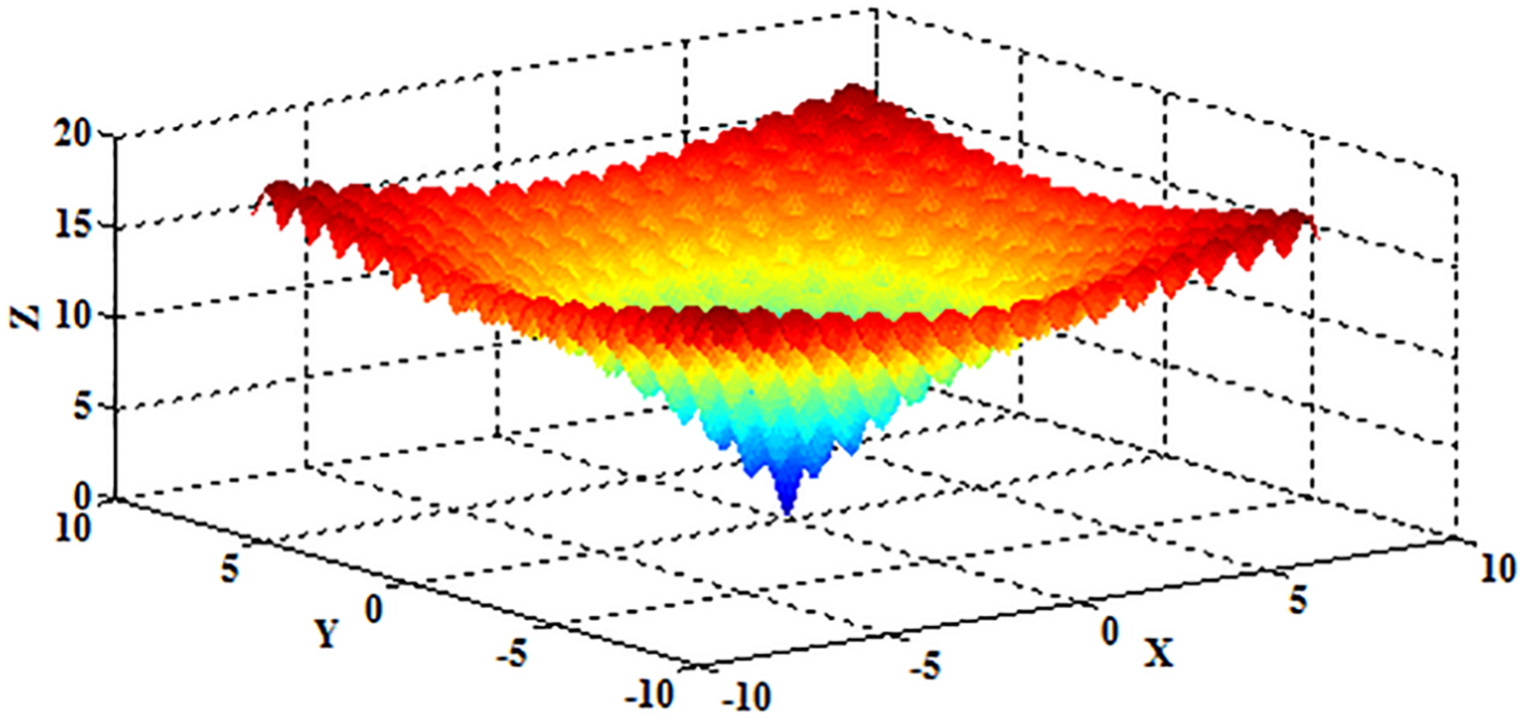
The schematic diagram of the Ackley function.

The Ackley function is a continuous function that combines exponential function with moderately enlarged cosine function. The hook face is rolling and has a series of peaks and troughs [45]. It can be defined as

$$f_{3}\left(x\right)=-\text{20}\times e^{-0.2\sqrt{\frac{1}{n}\sum_{i=1}^{n}x_{i}{}^{2}}}-e^{\frac{1}{n}\sum_{i=1}^{n}\text{cos}\left(2\pi x_{i}\right)}+20+e,$$ (21)

where *x*_*i*_∈[−32.768,32.768], and *D* represents the dimension of the independent variable *x*. The schematic diagram is shown in Fig 21 when *D* = 2.

As shown in Fig 21, the Ackley function is multimodal, and the optimum value 0 can be obtained when (*x*_1_,*x*_2_,⋯,*x*_*D*_) = (0,0,⋯,0).

**Fig 22:**
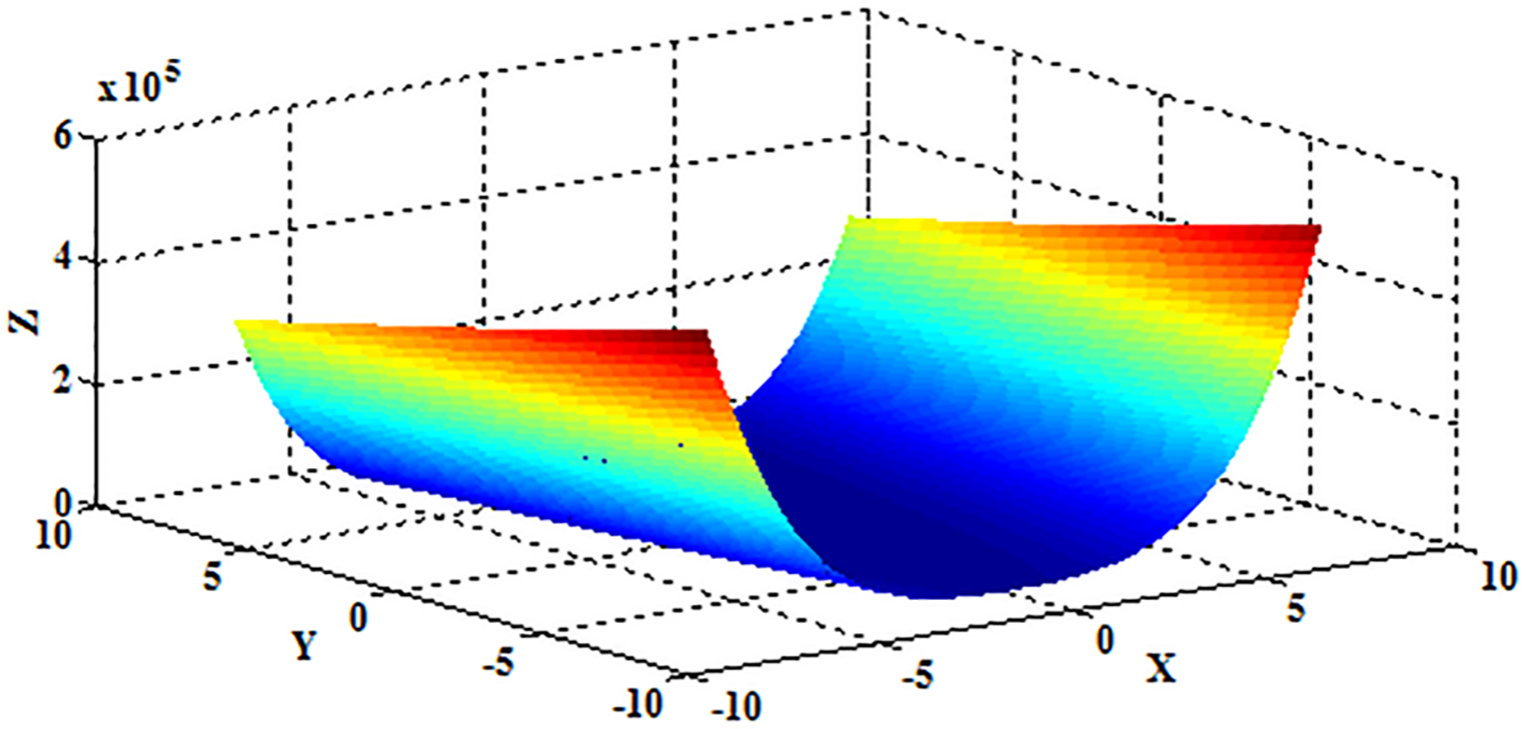
The schematic diagram of the Rosenbrock function.

The Rosenbrock function has significant interactions between variables. The global minimum is inside a long, narrow, parabolic-shaped flat valley. Although the valley does not have local minima, it is still difficult to find the global optimum. Hence, this function has been repeatedly used to assess the performance of optimization algorithms [44]. It is represented by

$$f_{4}\left(x\right)=\sum_{i=1}^{n-1}\left[100{\left(x_{i+1}-x_{i}{}^{2}\right)}^{2}+{\left(x_{i}-1\right)}^{2}\right],$$ (22)

where *x*_*i*_∈[−2.048,2.048], and *D* represents the dimension of the independent variable *x*. The schematic diagram is shown in Fig 22 when *D* = 2.

From Fig 22, the Rosenbrock function is unimodal, and the optimum value 0 can be obtained when (*x*_1_,*x*_2_,⋯,*x*_*D*_) = (0,0,⋯,0).

Table 11 shows the test results of the benchmark functions including the average convergence values of 10 experiments in different population sizes. The relevant parameters are as follows: the global maximum number of iterations is 200, the local maximum number of iterations is 20, the number of sub-populations is 5, and the dimension is 10.

**Table 11:** Average convergence values of benchmark functions.

Benchmark function	Population size	QSFLA-NSM	QSFLA	SFLA	QPSO	PSO	QGA	GA
Griewank	10	0.2516	1.3486	0.4904	5.8214	6.2649	3.5945	12.0721
	20	0.2571	0.8916	0.5305	0.7781	1.7089	1.6660	12.7720
	30	0.2778	0.4473	0.3142	1.2514	1.3703	1.2875	16.1515
Rastrigrin	10	14.9302	16.9191	23.0450	26.5873	38.2349	30.8697	32.1025
	20	11.9395	12.8997	12.3715	26.6794	32.7331	24.6563	30.7120
	30	10.3476	10.0336	12.8244	12.1873	18.7523	18.4079	31.6816
Ackley	10	0.4954	0.8580	2.3263	11.9030	16.7520	7.4565	5.9343
	20	0.2318	0.3894	2.6033	12.2852	7.7931	4.5118	6.7078
	30	0.0325	0.0809	2.0629	9.9039	2.8736	3.8106	6.1702
Rosenbrock	10	3.1191	13.5319	8.5369	19.1563	12.2326	22.1763	137.7837
	20	2.7361	9.4866	8.3268	15.3731	9.2875	17.0752	138.7428
	30	2.8610	9.2017	8.6349	11.3338	8.6008	9.2916	93.6943

As shown in Table 11, the average convergence values of the proposed QSFLA-NSM are closer to the optimum value 0. Therefore, the searching ability of the proposed QSFLA-NSM is better than the other intelligent optimization algorithms.

Table 12 shows the average time complexity (s) for the 10 experiments on the benchmark functions.

**Table 12:** Average time complexity of 10 experiments (s).

Benchmark function	Population size	QSFLA-NSM	QSFLA	SFLA	QPSO	PSO	QGA	GA
Griewank	10	0.65	34.48	0.43	0.03	0.02	0.41	0.03
	20	0.74	75.28	0.57	0.05	0.04	0.79	0.05
	30	0.81	108.88	0.62	0.07	0.06	1.16	0.08
Rastrigrin	10	0.48	32.93	0.32	0.02	0.02	0.39	0.02
	20	0.58	70.32	0.41	0.03	0.03	0.78	0.05
	30	0.67	109.34	0.51	0.05	0.04	1.14	0.06
Ackley	10	0.73	39.83	0.74	0.03	0.03	0.55	0.04
	20	0.85	87.81	0.83	0.08	0.06	1.09	0.08
	30	0.93	131.07	0.97	0.12	0.08	1.59	0.13
Rosenbrock	10	0.62	43.63	0.43	0.02	0.01	0.51	0.02
	20	0.77	93.77	0.56	0.04	0.03	1.04	0.05
	30	0.91	139.01	0.71	0.12	0.04	1.54	0.08

As depicted in Table 12, the average time complexity of QSFLA is the highest in among all the algorithms. However, the proposed QSFLA-NSM takes much less time to obtain accurate convergence values.

In fact, the frog population is encoded by quantum bits in QSFLA, which needs to be transformed into binary sequences by a decoding process. Then, the fitness function value of each frog individual is computed by the resulting decimal numbers. When the dimension of the solution space is higher, the encoding length of each frog individual is longer. Correspondingly, the time complexity is higher. However, each frog individual is directly encoded by real numbers in the proposed QSFLA-NSM, so the time complexity is significantly reduced. Meanwhile, because all the frog individuals in each sub-population participate in the local search to enhance the searching ability in QSFLA, the running time increases exponentially. But the proposed QSFLA-NSM needs to update only the worst frog in the sub-population, so the time complexity is further reduced to some extent in this paper.

For underwater sonar image detection, the number of clustering centers is 3, which represents the object-highlight region, the shadow region and the background region. Meanwhile, each clustering center contains only one property, namely the gray value. Therefore, the dimension of the solution space is 3. In addition, the population size is 12, and the number of sub-populations is 6, so there are only two frog individuals in every sub-population. Although the time complexity becomes higher, the magnitude of increase is not too large. For the experiments on the UCI data sets and the benchmark functions, the dimensions of solution spaces are both high, which makes the encoding length of QSFLA become very long. Additionally, there are more frog individuals in every sub-population. Therefore, the time complexity of QSFLA becomes very high.

From the all comparative experiments above, the proposed novel denoising method avoids the disadvantages of the filtering degree parameter in non-local spatial information. It can effectively remove noise and is conducive to the subsequent detection of underwater objects as shown in Figs 4(a), 5(d), 6(d), 16(b), 17(b) and 18(b). In QSFLA-NSM, each frog individual is directly encoded by real numbers to greatly simplify the evolution process. Moreover, a new search mechanism is developed in this paper to improve the searching ability and detection accuracy of sonar image as shown in Figs 7(a), 10(a), 11(a), 16(c), 17(c), and 18(c). The proposed QSFLA-NSM reduces time complexity and achieves a relatively higher detection accuracy than other intelligent optimization algorithms as shown in Figs 7–13. Meanwhile, the detection results of QSFLA-NSM are also closer to the global optimal solution as shown in Tables 4–6, Figs 9, 14 and 15. Furthermore, the results on the UCI data sets and the benchmark functions further demonstrate that the proposed method is more adaptable in Tables 8, 9, 11 and 12. Therefore, the proposed model has a certain effectiveness and adaptability that can detect original sonar images with floating objects, partly buried objects and objects on the bottom. The proposed method can also provide better preconditions for the further feature extraction and underwater object recognition.

## Conclusions

Detection accuracy and time complexity are both important factors for measuring the quality of object detection in underwater sonar images. QSFLA is a promising method, but because serious noise exists in the sonar image, the model performance is significantly reduced and cannot meet the accuracy requirements in the evolution process. In contrast, a novel denoising method is proposed to effectively remove noise, which greatly improves the detection performance. Then, to greatly simplify the evolution process, each frog individual is directly encoded by real numbers in the proposed QSFLA-NSM, and a fitness function combining intra-class difference with inter-class difference is adopted to evaluate the frog positions more accurately. Finally, a new search mechanism is developed in this paper to improve the searching ability and detection accuracy of sonar image. Meanwhile, the QSFLA-NSM also further reduces the time complexity to obtain accurate detection results more quickly.

We applied the proposed method to the original sonar images, the UCI data sets and the benchmark functions, and results show that the proposed method has better effectiveness and adaptability.
